# Elevated Proteasome Capacity Extends Replicative Lifespan in *Saccharomyces cerevisiae*

**DOI:** 10.1371/journal.pgen.1002253

**Published:** 2011-09-08

**Authors:** Undine Kruegel, Brett Robison, Thomas Dange, Günther Kahlert, Joe R. Delaney, Soumya Kotireddy, Mitsuhiro Tsuchiya, Scott Tsuchiyama, Christopher J. Murakami, Jennifer Schleit, George Sutphin, Daniel Carr, Krisztina Tar, Gunnar Dittmar, Matt Kaeberlein, Brian K. Kennedy, Marion Schmidt

**Affiliations:** 1 Department of Biochemistry, Albert Einstein College of Medicine, New York, New York, United States of America; 2 Department of Biochemistry, University of Washington, Seattle, Washington, United States of America; 3 Buck Institute, Novato, California, United States of America; 4 Max Delbrueck Center for Molecular Medicine, Berlin, Germany; 5 Department of Pathology, University of Washington, Seattle, Washington, United States of America; 6 Department of Molecular and Cellular Biology Program, University of Washington, Seattle, Washington, United States of America; Stanford University Medical Center, United States of America

## Abstract

Aging is characterized by the accumulation of damaged cellular macromolecules caused by declining repair and elimination pathways. An integral component employed by cells to counter toxic protein aggregates is the conserved ubiquitin/proteasome system (UPS). Previous studies have described an age-dependent decline of proteasomal function and increased longevity correlates with sustained proteasome capacity in centenarians and in naked mole rats, a long-lived rodent. Proof for a direct impact of enhanced proteasome function on longevity, however, is still lacking. To determine the importance of proteasome function in yeast aging, we established a method to modulate UPS capacity by manipulating levels of the UPS–related transcription factor Rpn4. While cells lacking *RPN4* exhibit a decreased non-adaptable proteasome pool, loss of *UBR2*, an ubiquitin ligase that regulates Rpn4 turnover, results in elevated Rpn4 levels, which upregulates UPS components. Increased UPS capacity significantly enhances replicative lifespan (RLS) and resistance to proteotoxic stress, while reduced UPS capacity has opposing consequences. Despite tight transcriptional co-regulation of the UPS and oxidative detoxification systems, the impact of proteasome capacity on lifespan is independent of the latter, since elimination of Yap1, a key regulator of the oxidative stress response, does not affect lifespan extension of cells with higher proteasome capacity. Moreover, since elevated proteasome capacity results in improved clearance of toxic huntingtin fragments in a yeast model for neurodegenerative diseases, we speculate that the observed lifespan extension originates from prolonged elimination of damaged proteins in old mother cells. Epistasis analyses indicate that proteasome-mediated modulation of lifespan is at least partially distinct from dietary restriction, Tor1, and Sir2. These findings demonstrate that UPS capacity determines yeast RLS by a mechanism that is distinct from known longevity pathways and raise the possibility that interventions to promote enhanced proteasome function will have beneficial effects on longevity and age-related disease in humans.

## Author Summary

The ubiquitin/proteasome system (UPS) is an integral part of the machinery that maintains cellular protein homeostasis and represents the major pathway for specific protein degradation in the cytoplasm and nuclei of eukaryotic cells. Its proteolytic capacity declines with age. In parallel, substrate load for the UPS increases in aging cells due to accumulated protein damage. This imbalance is thought to be an origin for the frequently observed accumulation of protein aggregates in aged cells and is thought to contribute to age-related cellular dysfunction. In this study, we investigated the impact of proteasome capacity on replicative lifespan in *Saccharomyces cerevisiae* using a genetic system that allows manipulation of UPS abundance at the transcriptional level. The results obtained reveal a positive correlation between proteasome capacity and longevity, with reduced lifespan in cells with low proteasome abundance or activity and strong lifespan extension upon up-regulation of the UPS in a mechanism that is at least partially independent of known yeast longevity modulating pathways. The same correlation is observed for oxidative and protein stress tolerance and clearance of toxic huntingtin fragments in a yeast model for neurodegenerative diseases, suggesting that lifespan extension by increased proteasome capacity is caused by improved protein homeostasis.

## Introduction

Oxidative and other forms of damage to cellular components occur throughout the lifespan of organisms [1]. Young cells are protected by efficient repair and elimination systems, while aging cells gradually accumulate damaged macromolecules and organelles leading eventually to a catastrophic functional deterioration and cell death. Many reports document a gradual decline in repair and maintenance systems in aging cells [2], suggesting that impaired repair and clearance of damaged macromolecules represents a crucial origin of age-related cellular dysfunction [3].

Ribosomes, chaperones and two proteolytic systems, the proteasome and the autophagosomal/lysosomal pathway, are responsible for the maintenance of protein homeostasis [4]. If translation and chaperone-assisted protein folding/disaggregation fail, larger protein aggregates or damaged organelles are cleared by macroautophagy in all eukaryotes, while specific proteins are removed from the cytoplasm by chaperone-mediated autophagy in mammals [5]. Both activities result in destruction of damaged molecules in an enclosed hydrolytic environment, the lysosome. Damaged proteins in the nucleus and cytoplasm of eukaryotic cells are eliminated by the ubiquitin/proteasome system [6].

The proteasome is a highly conserved, multicatalytic enzyme composed of more than 33 subunits, which form two entities. The central proteolytic core (CP) contains the proteolytically active sites sequestered within an enclosed cylinder, while a regulatory complex (RP) mediates substrate recognition, processing and transport into the catalytic chamber of the CP [6]. Crucial components of the RP are six paralogous ATPases. They actively unfold bound proteins, a process that is required for the passage of substrates through a narrow gate leading into the interior of the CP [6], [7]. Substrate binding by the proteasome requires covalent modification of a target protein with a polyubiquitin chain, which is attached to a substrate via a three-step enzymatic cascade involving ubiquitin activation by E1 enzymes, ubiquitin conjugation by E2 proteins and covalent linkage of ubiquitin to substrates by E3 ubiquitin ligases [6].

Many studies demonstrate a decline in proteasome function with age in different organisms [8], [9]. Proteasome impairment is reported at several levels, including decreased transcription of certain proteasomal subunits in mice [10], dissociation of the holocomplex in *Drosophila* [11], and reduced proteolytic capacity in different aged mammalian tissues and organs [12]. In contrast, centenarians and the long-lived naked mole rats exhibit elevated proteasome levels and activity [13], [14]. Initial genetic approaches suggest a role for proteasome function in modulating longevity. In the fission yeast *Saccharomyces pombe*, the maintenance of a quiescent G0 state is dependent on functional proteasomes [15], and downregulation of proteasome RP subunits in *C. elegans* leads to a profound shortening of lifespan [16]. Moreover, studies in *Saccharomyces cerevisiae* and *Drosophila* indicate that overexpression of proteasome-related genes might exert a positive effect on lifespan. Elevated levels of the proteasome biogenesis factor Ump1 are reported to extend yeast stationary phase survival [17], although replicative lifespan has not been investigated. Furthermore, overexpression of the proteasomal deubiquitinating subunit Rpn11 extends lifespan in flies [18]. While enhanced proteasome activity was speculated to be the cause of lifespan extension in both studies, the underlying impact of UPS component overexpression on proteasome biology was not fully elucidated. Studies in yeast suggest a proteasome independent function of a free Rpn11 pool in the maintenance of mitochondrial morphology, thus extended lifespan in flies overexpressing Rpn11 might result from functions of Rpn11 that are independent of the proteasome [19].

Here we present a genetically defined system utilizing transcriptional regulation of the UPS to study the effect of decreased and elevated proteasome capacity on yeast replicative lifespan. Expression of UPS genes in yeast and mammals is regulated by a feedback mechanism [20], [21]. In the absence of proteotoxic stress, the UPS-related transcription factor Rpn4 has a short half-life of several minutes, being rapidly ubiquitinated by the E3 ligase Ubr2 and degraded by the proteasome [22]. This feedback loop ensures a fast and tightly regulated increase in proteasome levels during protein stress; increased degradation of proteasome substrates reduces turnover of Rpn4, promoting a compensatory elevation of proteasomal gene transcription [20], [21]. Cells deleted for *RPN4* exhibit reduced basal non-adaptable proteasome levels. In contrast, loss of the E3 ligase Ubr2 leads to Rpn4 stabilization, which in turn results in elevated proteasome abundance and increased UPS capacity [22]. Utilizing this system, we found that proteasome abundance correlated with longevity. Deletion of *RPN4* shortened lifespan, while loss of *UBR2* dramatically increased lifespan, demonstrating a beneficial effect of elevated proteasome function on cell physiology. The same correlation between reduced and elevated proteasome capacity is evident from phenotype analysis in the presence of proteotoxic stress. Increased turnover of structurally unstable proteins as well as enhanced clearance of aggregation prone huntingtin fragments was observed in cells with elevated proteasome function, whereas loss of *UBR2* failed to induce lifespan extension in cells with compromised proteasome function. These findings argue for a model in which the beneficial effect of increased proteasome capacity on longevity is related to improved elimination of damaged or aggregation prone proteins and thus improved proteostasis, possibly representing a major function of the proteasomes in aging cells.

## Results

### Reduced replicative lifespan in proteasome hypomorphic mutants

**Figure 1:**
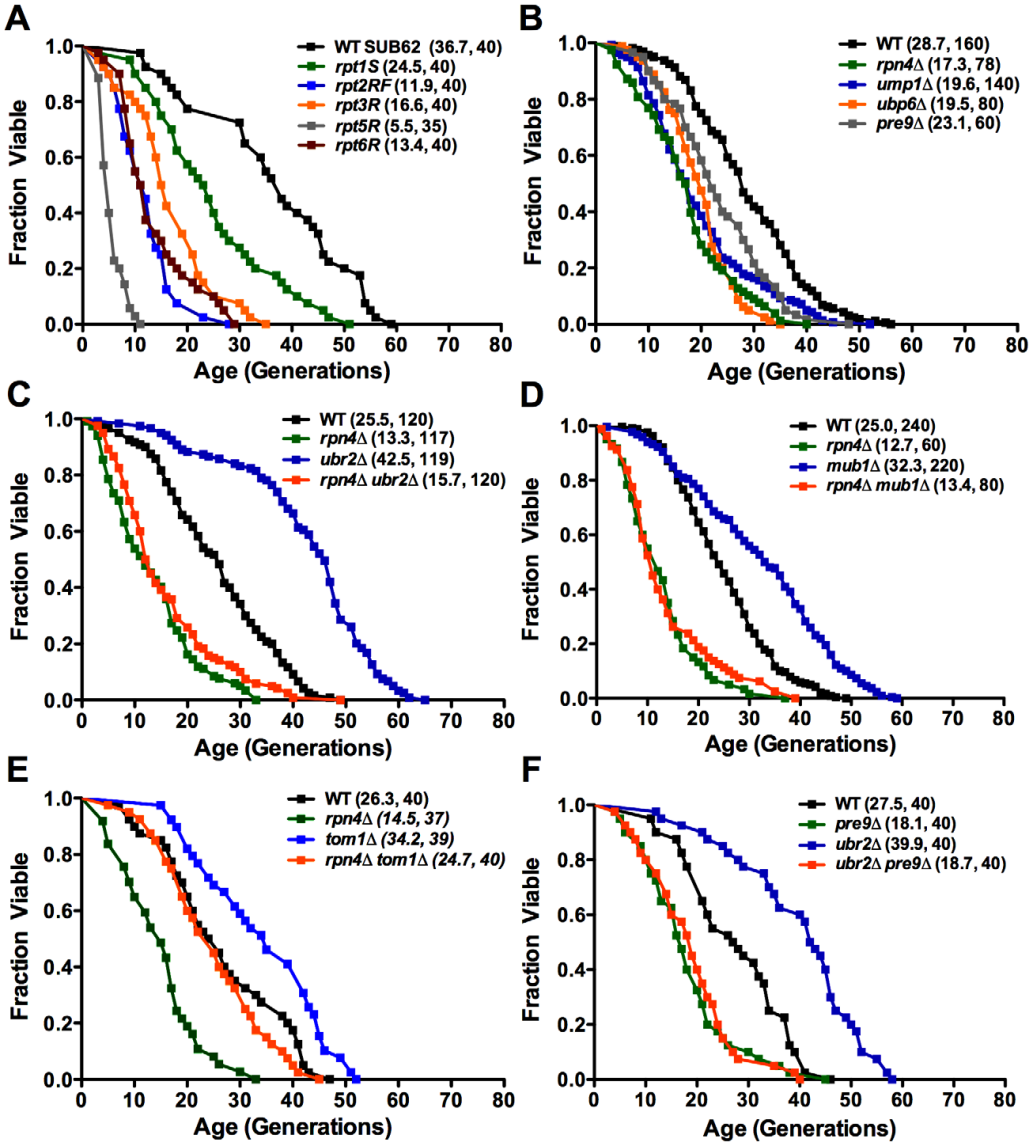
Replicative lifespan in cells with proteasome-related gene mutations or deletions. Mean lifespan and cell counts are shown in parenthesis. (A) Proteasome ATPase mutants. Survival curves for cells with individually inactivated proteasomal ATPases (SUB62 strain background). (B) Viable proteasome-related gene deletions. Survival curves for cells deleted for the proteasome maturation factor *UMP1*, the proteasome associated deubiquitinating enzyme *UBP6,* the only non-essential CP subunit *PRE9*, and the proteasome-related transcription factor *RPN4* (BY strain background). (C) and (D) RLS lifespan extension in *ubr2Δ* (C) and *mub1Δ* (D) cells. Survival curves of cells deleted for *RPN4, UBR2*, or *RPN4 UBR2* (C) or *RPN4, MUB1*, or *RPN4 MUB1* (D). (E) Deletion of *TOM1* causes RLS extension independent of *RPN4*. Survival curves of cells deleted for *RPN4, TOM1,* or *RPN4 TOM1*. (F) Lifespan extension induced by loss of *UBR2* requires functional proteasomes. Survival curves of cells deleted for *PRE9, UBR2,* or *PRE9 UBR2*. A statistical analysis of the data is summarized in Table S2.

To more broadly define the importance of proteasome function in aging, we measured replicative lifespan (RLS) for several yeast mutants that recapitulate the individual processes causing proteasome dysfunction in mammalian aging and cell senescence: loss of activity, impaired assembly/structural integrity and decreased abundance [8], [9]. The proteasomal ATPases Rpt1-6 are essential components of the catalytic cycle of the proteasome. Mutants with inactivated ATP-hydrolysis are viable but show impaired substrate turnover [23]. Consistent with the observed decline in proteolytic capacity of the proteasome with aging in multicellular eukaryotes, ATPase mutants in 5 of these 6 components exhibited reduced RLS (Figure 1A). The varying degree in RLS reduction is in agreement with previous findings that the ATPases play non-redundant roles in proteasome-mediated substrate turnover [23]. Several further studies (Figure 1B) emphasize the requirement of normal proteasome function for normal lifespan. First, deletion of the proteasome biogenesis factor *UMP1*, which is required for correct proteasome maturation [24], leads to reduced RLS. Second, deletion of the only non-essential integral proteasome core subunit *PRE9*, which results in impaired proteasome assembly and impaired proteolytic capacity [25], shortens RLS. Third, a *ubp6Δ* strain is also short-lived. Ubp6 is a proteasome associated deubiquitinating enzyme, which influences ubiquitin chain length of proteasome-bound substrates and thereby impacts the turnover of proteasome substrates [26]. Lastly, cells lacking the transcriptional regulator of proteasome expression, the C_2_H_2_ zinc-finger transcription factor *RPN4*, which are characterized by a reduced, non-adaptable proteasome pool [20], [21], exhibit a 40% reduction in mean RLS (Figure 1B). These results demonstrate that defects in several different aspects of proteasome biology are sufficient to shorten lifespan.

### Increased replicative lifespan in cells with elevated proteasome capacity

Since reduction in proteasome function resulted in decreased RLS, we reasoned that an increase in proteasome number might enhance longevity. An ideal candidate to manipulate proteasome levels is Rpn4, due to its specific function as a regulator of proteasomal gene transcription [20], [21]. We hypothesized that a rise in Rpn4 protein levels would increase proteasome abundance and might positively impact lifespan. Loss of either of two proteins is known to result in elevated Rpn4 protein levels: the E3 ubiquitin ligase Ubr2 [22], and Mub1, a protein required for Ubr2-mediated Rpn4 ubiquitination [27]. To test whether elevated Rpn4 levels enhance longevity, we determined the RLS of strains lacking *UBR2* or *MUB1*. Strikingly, both *ubr2Δ* and *mub1Δ* mother cells were significantly longer-lived than wild type cells (Figure 1C, 1D). This effect was dependent on Rpn4, since deletion of *RPN4* abrogated lifespan extension in *ubr2Δ* or *mub1Δ* strains (Figure 1C, 1D), arguing for a model in which the enhanced longevity of *ubr2Δ* and *mub1Δ*cells resulted from stabilization of Rpn4. Importantly, *RPN4* is not required for lifespan extension in a long-lived strain lacking *TOM1* (Figure 1E), another E3 ligase involved in transcriptional regulation [28]. These observations demonstrate that lifespan extension by Rpn4 stabilization is specific for *UBR2* and *MUB1*. Rpn4 stabilization is necessary but not sufficient for a positive effect on lifespan, since compromised proteasome function, induced by loss of *PRE9*, prevents lifespan extension in *ubr2Δ* cells (Figure 1F).

**Figure 2:**
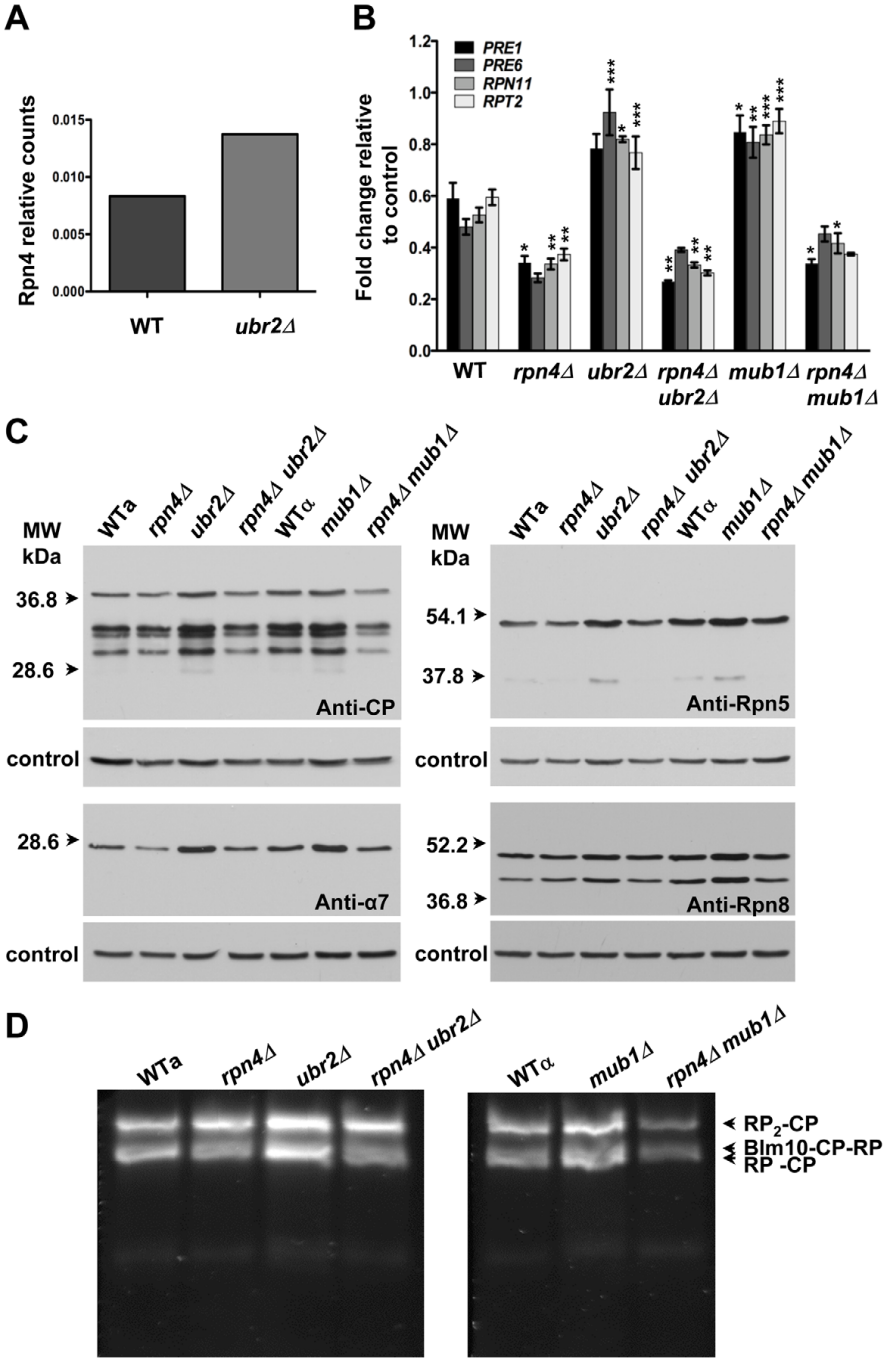
Extended proteasome biogenesis and subunit abundance upon loss of *UBR2* or *MUB1.* (A) Relative quantification of Rpn4 abundance in WT and *ubr2Δ* cells via SRM (selected reaction monitoring) (B) Expression of four proteasomal genes in cells deleted for *RPN4*, *UBR2, MUB1* or *RPN4 UBR2* and *RPN4 MUB1.* The mRNA levels of two genes encoding proteasome CP subunits (*PRE1* and *PRE6*) or RP subunits (*RPN11* and *RPT2*) were tested by qRT-PCR and presented relative to the housekeeping gene *PRP8* as the mean +/− SEM of three independent experiments. *P*-values represent the statistical significance relative to WT gene expression. *  =  *P*-value of <0.05 **  =  *P*-value of <0.01 ***  =  *P*-value of <0.001. A complete table of *P*-values can be found in Figure S1. (C) Elevated proteasome subunit levels in *ubr2Δ* and *mub1Δ*. Normalized lysates of the strains indicated were subjected to SDS-PAGE and immuno-blotting with antibodies directed against CP (anti-CP and anti-α7) or RP subunits (anti-Rpn8 and anti-Rpn5). The position of marker proteins is indicated to the left. (D) Unfractionated lysates of the strains indicated were subjected to native gel electrophoresis followed by an in-gel activity assay with the fluorogenic proteasome-specific substrate Suc-LLVY-AMC. The fluorogenic signals shown represent proteasome holocomplex activity. RP_2_-CP denotes proteasome core particles (CP) flanked on both sides with regulatory particles (RP), Blm10-CP-RP a proteasome hybrid complex with the CP flanked on one side with the proteasome activator Blm10 and on the other side with RP, RP-CP a singly RP capped CP.

**Figure 3:**
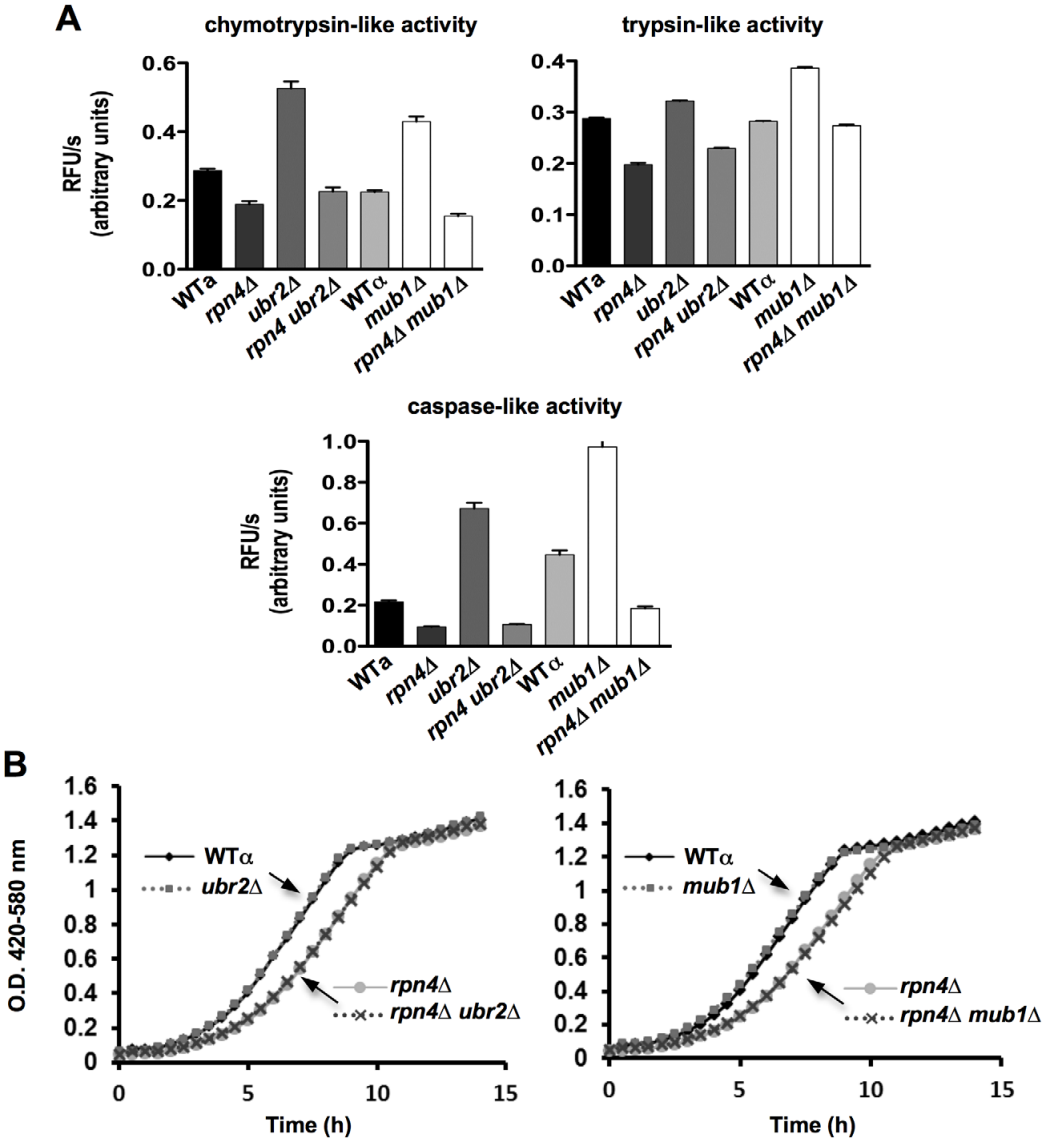
Elevated peptidase activity of proteasome holocomplexes in *ubr2Δ* and *mub1Δ* cells. (A) Elevated cellular proteasomal peptidase activity in *ubr2Δ* and *mub1Δ* strains. The three distinct proteasomal peptidase activities of the strains indicated were tested with 50 µg of total protein in the presence of 100 µM of the fluorogenic substrates Suc-LLVY-AMC (chymotrypsin-like activity), Boc-RLR-AMC (trypsin-like activity) and Ac-nLPnLD-AMC (caspase activity). The reactions were recorded in the absence or presence of 50 µg/ml of the proteasome inhibitor MG132 (Figure S2) to demonstrate specificity of the reactions. The mean +/− SEM of three technical replicates is presented. (B) Growth is unaffected in cells with increased proteasome abundance. Growth curves of WT, *rpn4Δ, ubr2Δ, mub1Δ, rpn4Δ ubr2Δ *and* rpn4Δ mub1Δ* strains were recorded in a Bioscreen C MB machine in YPD at 30°C. **Doubling times are listed in Figure S3.

Consistent with increased Rpn4 abundance in cells lacking *UBR2* (Figure 2A) or *MUB1* we observed increased transcription of proteasomal genes, both for components of the RP (*RPN11* and *RPT2)* and the CP (*PRE1* and *PRE6*) (Figure 2B). The increased transcription of proteasomal genes resulted in elevated protein levels of RP and CP subunits (Figure 2C). To verify that the increased subunit production leads to increased abundance of functional proteasome complexes, we visualized active proteasome populations in unfractionated lysates (Figure 2D) and tested the three distinct proteasomal peptidase activities with proteasome-specific fluorogenic peptide substrates from lysates of *ubr2Δ* or *mub1Δ*cells (Figure 3A, Figure S2). Both assays showed increased proteasome capacity upon Rpn4 stabilization. Growth of the strains remained, however, largely unaffected (Figure 3B, Figure S3). In contrast, loss of *RPN4* showed the opposite characteristics (Figure 2, Figure 3A) and resulted in reduced growth rate (Figure 3B, Figure S3). In every case, a direct correlation between proteasome capacity and lifespan was observed: cells with a reduced proteasome pool (*rpn4Δ, rpn4Δ ubr2Δ rpn4Δ mub1Δ*) exhibited shortened RLS (Figure 1B–1E), while cells with high proteasome capacity (*ubr2Δ* or *mub1Δ*) showed increased median and maximum RLS, up to 70% longer than wild type cells (Figure 1C, 1D). This is comparable to the longest-lived single gene deletion strains reported or identified from our nearly complete screen of the yeast ORF deletion collection [29]–[31; data not shown].

### The oxidative stress response does not contribute to lifespan extension in cells with elevated Rpn4 levels

Damage to biological macromolecules resulting from reactive oxygen species is thought to represent a major cause for age-related cellular dysfunction. Two strategies are employed by cells to counteract oxidative damage: detoxification/repair and elimination. In yeast, both strategies are controlled at the transcriptional level via Rpn4, which regulates genes with PACE (proteasome associated control elements) promoter elements [32], and the AP-1 like leucine zipper transcription factor Yap1, the major regulator of the oxidative detoxification response [33]. Yap1 promotes expression of genes with YRE (Yap1 response elements) promoter elements. The regulation of both gene clusters is linked through a positive feed back loop between Rpn4 and Yap1. While Yap1 induces Rpn4 expression via an YRE element in the promoter of *RPN4*, Yap1 expression is induced by Rpn4 via a PACE element in the promoter of *YAP1* [34]. This bistability strategy most likely has evolved to ensure optimal response to oxidative stress. In cells deleted for *RPN4*, this feed back loop regulation causes reduced expression of Yap1 targets. Whether the inverse scenario, an upregulation of genes required for detoxification of reactive oxygen species (ROS), is observed upon stabilization of *RPN4* has not been investigated. Although lifespan extension induced by Rpn4 stabilization requires functional proteasomes (Figure 1F) a possible upregulation of Yap1 target genes in *ubr2Δ* and *mub1Δ* strains might contribute to the increased longevity exhibited by both strains.

**Figure 4:**
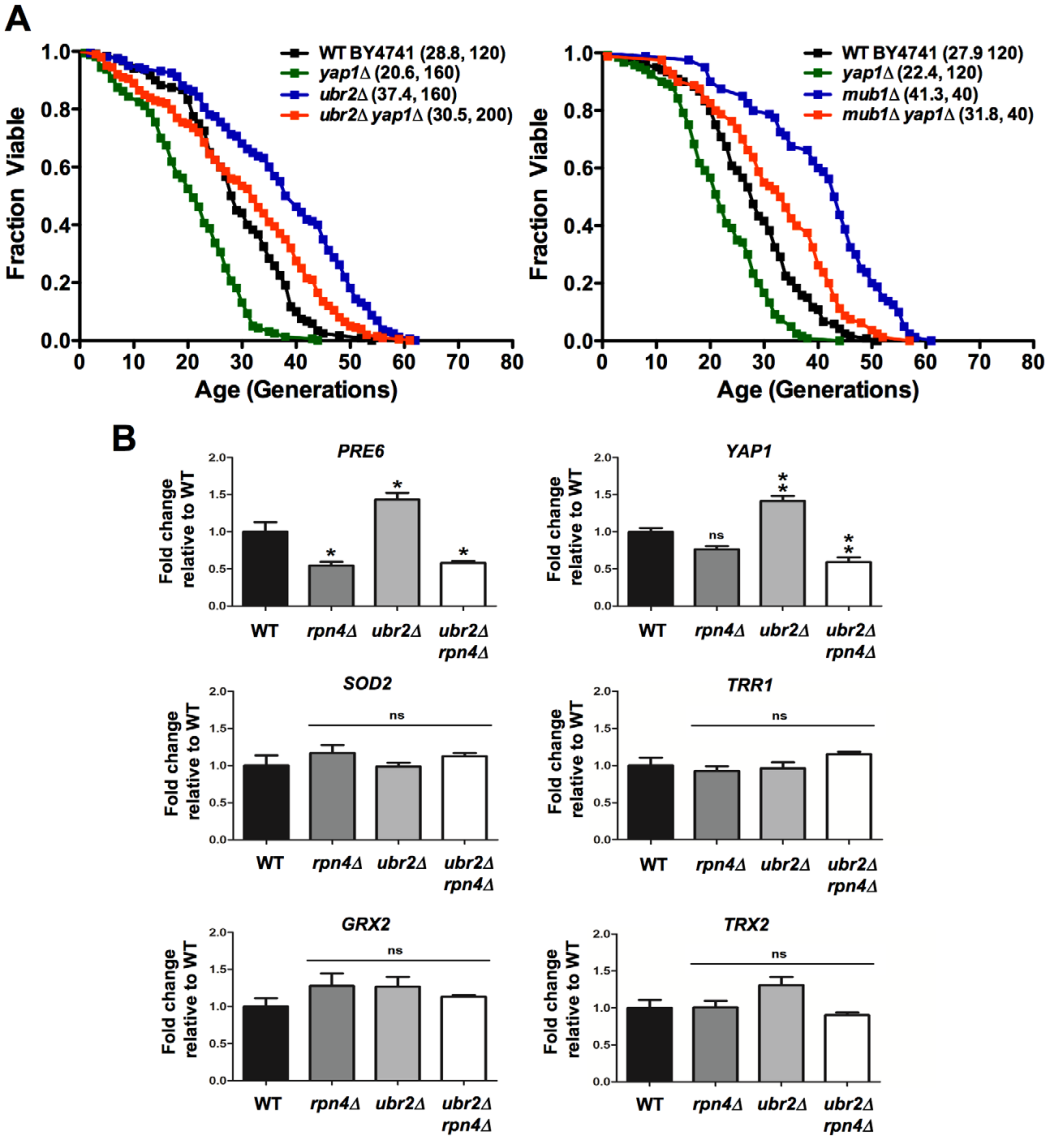
Proteasome-mediated lifespan extension is independent of the oxidative stress response. (A) Survival curves of cells deleted for *UBR2*, *YAP1* or *UBR2 YAP1* (left panel) or *MUB1, YAP1*, or *MUB1 YAP1* (right panel). Mean RLS and cell counts are shown in parenthesis. A statistical analysis of the data is summarized in Table S2. (B) Expression of Rpn4 target genes *PRE6* and *YAP1* as well as four Yap1 target genes *SOD2*, *TRR1*, *GRX2*, and *TRX2* in cells deleted for *RPN4*, *UBR2,* or *RPN4 UBR2.* The mRNA levels were tested by qRT-PCR and presented relative to the housekeeping gene *PRP8* as the mean +/− SEM of three independent experiments. *P*-values represent the statistical significance relative to WT gene expression. *  =  *P*-value of <0.05, **  =  *P*-value of <0.01, ns  =  not significant.

**Figure 5:**
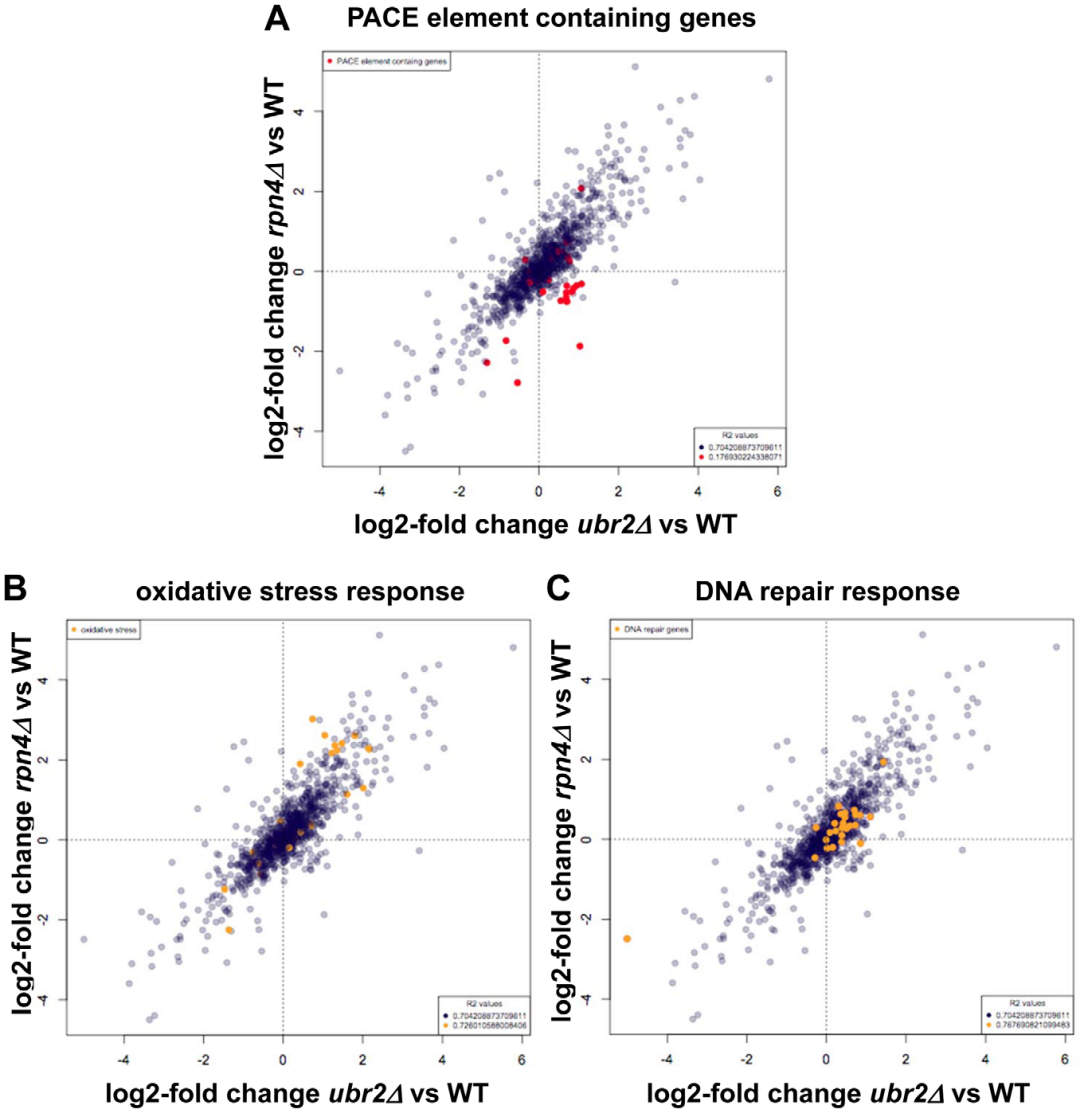
SILAC–based quantitative large-scale proteomics of cells with reduced and increased proteasome capacity. (A) SILAC ratios for *rpn4Δ*/WT were plotted on the y-axis and SILAC ratios for *ubr2Δ*/WT were plotted on the x-axis. (A) Proteins transcribed from genes containing the Rpn4 controlled PACE element were colored red. (B) Data points corresponding to proteins with the GO classification: oxidative stress (GO:0006979), were colored orange. (C) SILAC ratios were plotted as described in (A). Data points corresponding to proteins with the GO classification: DNA repair (GO:0006281) were colored orange. For all proteins and subselections Pearson correlation coefficients were calculated and R^2^ values are presented. Proteins with log2 >0.5 or <−0.5 fold expression changes are listed in Tables S3, S4, S5, S6.

To explore this possibility, we deleted *YAP1* in *ubr2Δ* and *mub1Δ* cells and examined their RLS. Loss of *UBR2* or *MUB1* (Figure 4A, right panel) extended the lifespan of *YAP1* deleted cells, providing evidence that Yap1 induced detoxification of ROS does not account for the increased longevity of cells with increased proteasome capacity. This conclusion is corroborated by the lack of induction of Yap1 target genes in *ubr2Δ*and *mub1Δ* cells (Figure 4B, middle and lower panels), despite upregulation of *YAP1* expression (Figure 4B, upper, right panel). The lack of Yap1 target gene induction is also apparent in a SILAC-based quantitative proteome analysis of *rpn4Δ* and *ubr2Δ*cells. While the protein levels of proteasome subunits correlate with Rpn4 abundance (reduced abundance in *rpn4Δ*, increased abundance in *ubr2Δ* cells) (Figure 5A, red labels), no correlation of proteins required for oxidative detoxification is observed (Figure 5B, yellow labels). The lack of increased Yap1 function despite elevated transcription of the gene may reflect additional mechanisms for controlling Yap1 activity, including proteasomal degradation [35] and regulated differential localization [36].

In addition to co-regulation of the UPS and the oxidative stress response, Rpn4 has been suggested to be involved in the induction of genes required for base-excision repair after treatment of cells with genotoxic agents [37], [38]. Our proteome data set, however, does not reveal a positive correlation between proteins involved in DNA repair and Rpn4 abundance (Figure 5C), thus it appears unlikely that upregulated DNA repair contributes to lifespan extension in cells with elevated Rpn4 levels.

### Improved proteotoxic stress response in cells with elevated proteasome capacity

**Figure 6:**
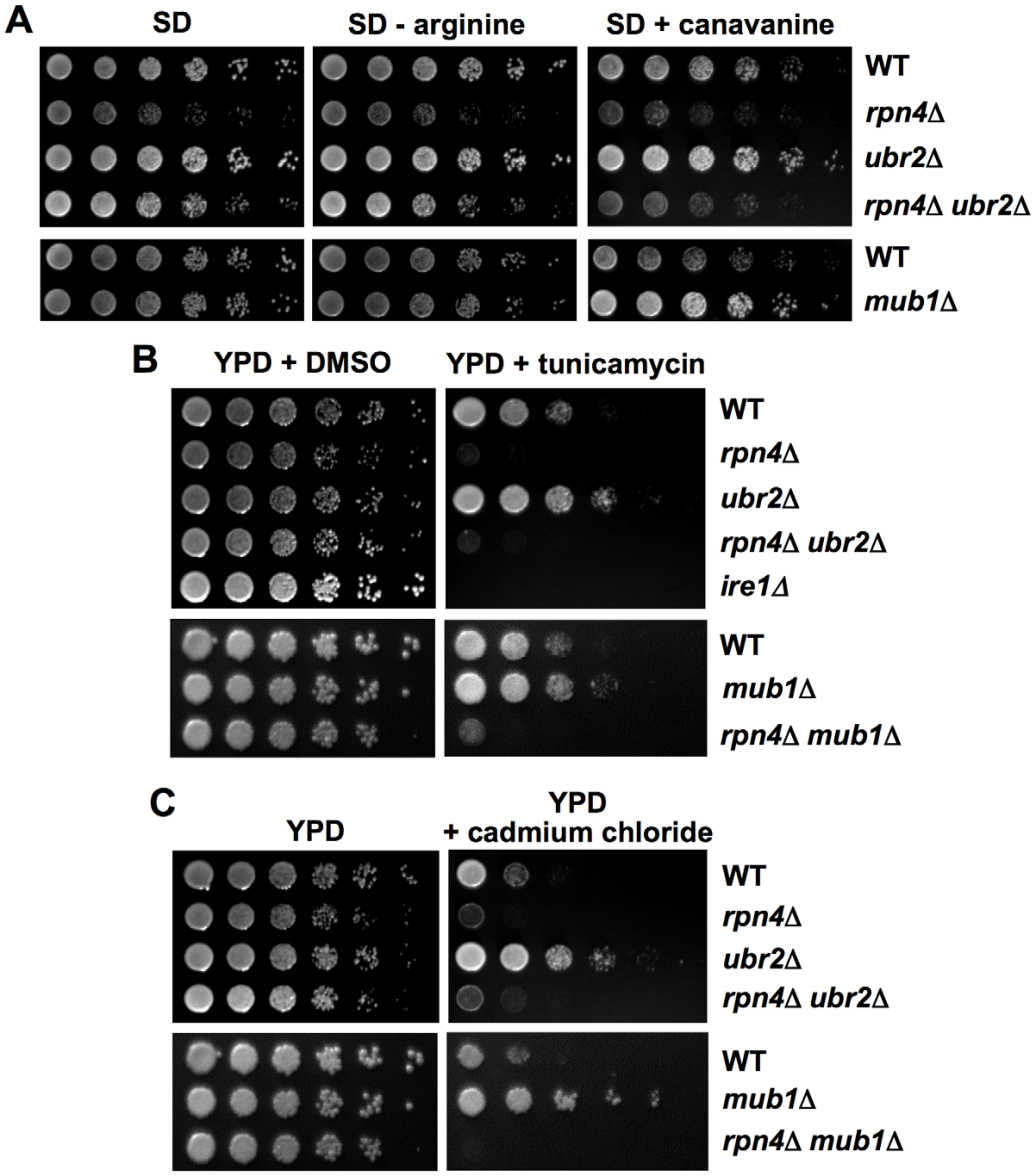
Correlation between resistance towards proteotoxic stress and proteasome abundance. Increased resistance to canavanine, ER or oxidative stress upon *UBR2* or *MUB1* deletion. Serially diluted log phase cultures of WT, *rpn4Δ, ubr2Δ, mub1Δ, rpn4Δ ubr2Δ *and* rpn4Δ mub1Δ* strains were spotted on (A) synthetic media in the presence (left panel) or absence (middle panel) of arginine, in the absence (left and middle panel) or presence (right panel) of 3 µg/ml of the arginine analog canavanine, on (B) complete media in the absence (left panel) or presence of 0.5 µg/ml tunicamycin (right panel) or on (C) complete media in the absence (left panel) or presence of 50 µM cadmium chloride (right panel).

To characterize the effect of elevated proteasome activity on protein homeostasis, we subjected the cells to several protein stress-inducing conditions, which require correct proteasome function for survival. Loss of *UBR2* or *MUB1* renders cells resistant to the proteotoxic arginine analog canavanine (Figure 6A), to ER stress as induced by tunicamycin (Figure 6B), and to the heavy metal cadmium, which inhibits enzymes involved in detoxification of reactive oxygen species (Figure 6C). In contrast, loss of *RPN4* results in profound sensitivity to exogenous protein stress (Figure 6). Importantly, *RPN4* is required for the stress resistance of *ubr2Δ* or *mub1Δ* cells, since a *ubr2Δ rpn4Δ* or a *mub1Δ rpn4Δ*strain is as sensitive as *rpn4Δ* in each of the three conditions. In accord, lifespan extension by *ubr2Δ* or *mub1Δ* is abrogated in a strain also lacking *RPN4* (Figure 1C, 1D). These findings indicate that Rpn4 stabilization, and thus elevated proteasome capacity, enhances the cell's ability to withstand proteotoxic stress and are consistent with the hypothesis that the increased lifespan of *ubr2Δ* or *mub1Δ* cells results directly from improved protein homeostasis.

### Improved elimination of unstable and aggregation prone proteins in cells with increased proteasome capacity

**Figure 7:**
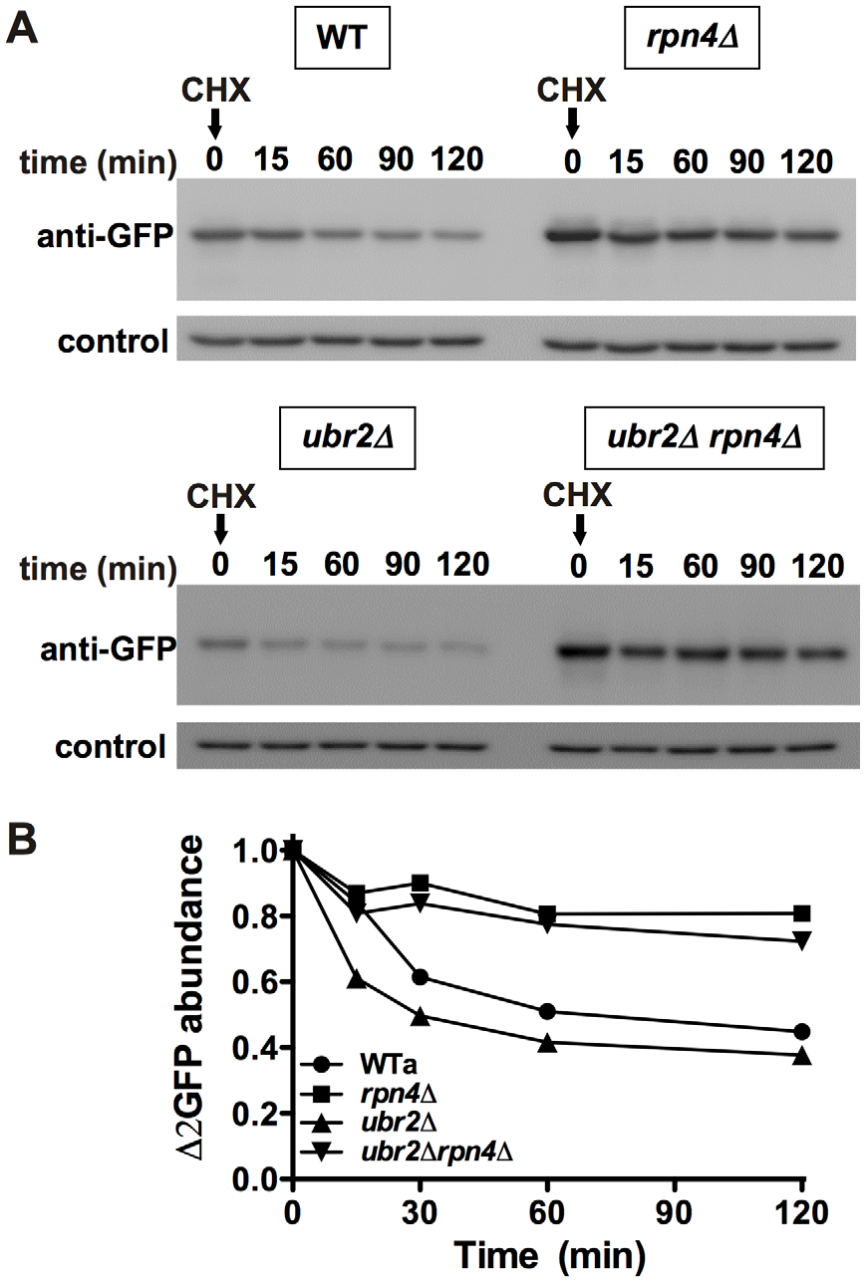
Cells with higher proteasome capacity exhibit increased turnover of unstable proteasome substrates *in vivo*. (A) The half-life of the unstable GFP variant *Δ*2GFP was determined in post diauxic shift WT (upper panel, left), *rpn4Δ *(upper panel, right), *ubr2Δ* (lower panel, left) and *ubr2Δ rpn4Δ* (lower panel, right) cells after blocking new synthesis with lethal doses of CHX, followed by immunodetection of the protein. anti-Pgk1 was used as a loading control (lower panels). (B) Signals in (A) were detected using ECL chemiluminescence in an ImageQuant instrument and quantified with the ImageQuantTL software. Control normalized values are presented.

**Figure 8:**
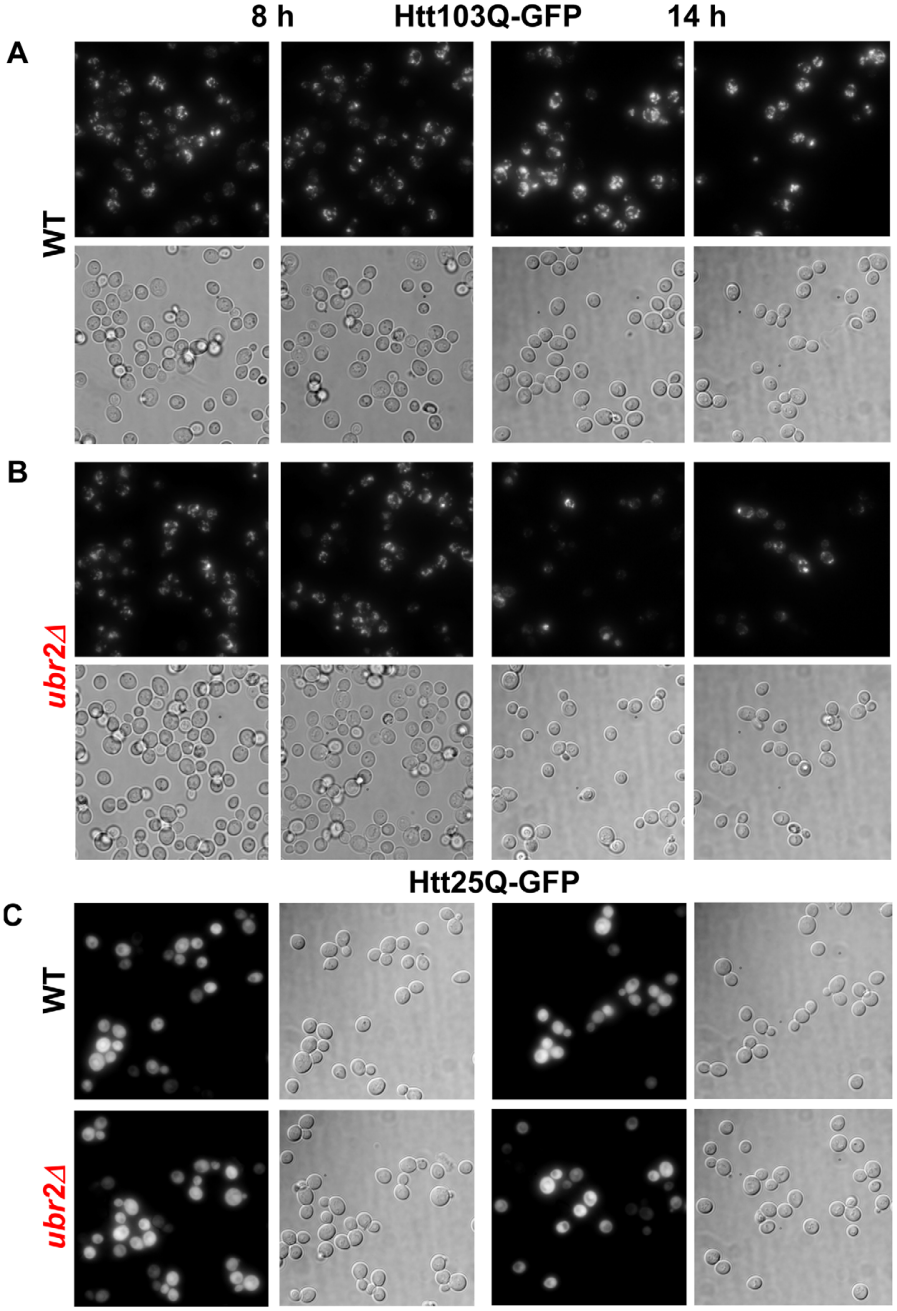
Amelioration of Htt103Q aggregation in cells with higher proteasome capacity. Live cell fluorescence images of WT (A) or *ubr2Δ* (B) cells expressing inducible Htt103Q-CFP or (C) Htt25Q-CFP were taken after 8 h (left panels) or 14 h of induction. 25Q overexpressing cells exhibit a soluble CFP signal after 8 h and 14 h of induction in WT and *UBR2* deleted cells. All fluorescent images were recorded under the same conditions and represent merged Z-stacks. Corresponding transmitted light images (DIC) are shown below each fluorescent image.

To test whether cells with increased proteasome capacity exhibit improved protein homeostasis, we investigated whether *ubr2Δ* cells exhibit improved clearance of unstable and aggregation prone proteins *in vivo*. First, we determined the degradation kinetics of a constitutively expressed unstable proteasome model substrate (*Δ*2GFP) [39] in cells with increased or reduced proteasome abundance after blocking new synthesis with lethal doses of cycloheximide (CHX). Accelerated degradation of *Δ*2GFP occurred in *ubr2Δ* cells, while *RPN4* deleted cells displayed reduced turnover (Figure 7A, 7B). The differential turnover rates in cells with varying proteasome levels is further reflected in the steady state level of *Δ*2GFP, as indicated by the amount of the protein present at time 0 (Figure 7A). Secondly, we tested a yeast model for age-associated neurodegenerative disorders in which inducible CFP is fused to the first exon of huntingtin with toxic (Htt103Q) or non-toxic (Htt25Q) glutamine expansions [40], [41]. These proteins were expressed in WT and *ubr2Δ* cell, and the abundance and cellular distribution of Huntingtin fragments were visualized by fluorescence in live cells (Figure 8). Previously, these constructs have been shown to form aggregates in yeast cells [40], [41]. After 8 hours of induction we did not observe a difference between WT (Figure 8A, left panel) and *ubr2Δ* cells. After 14 hours, however, the level of aggregates was strongly reduced in cells with elevated proteasome capacity (Figure 8B, right panel) as compared to WT cells (Figure 8A, right panel), suggesting improved clearance of the Htt aggregates. The cellular distribution and the abundance of a non-toxic huntingtin fragment, Htt25Q, remained unaffected by increased proteasome capacity (Figure 8C).

### Functional independence of proteasome-mediated effects on lifespan

**Figure 9:**
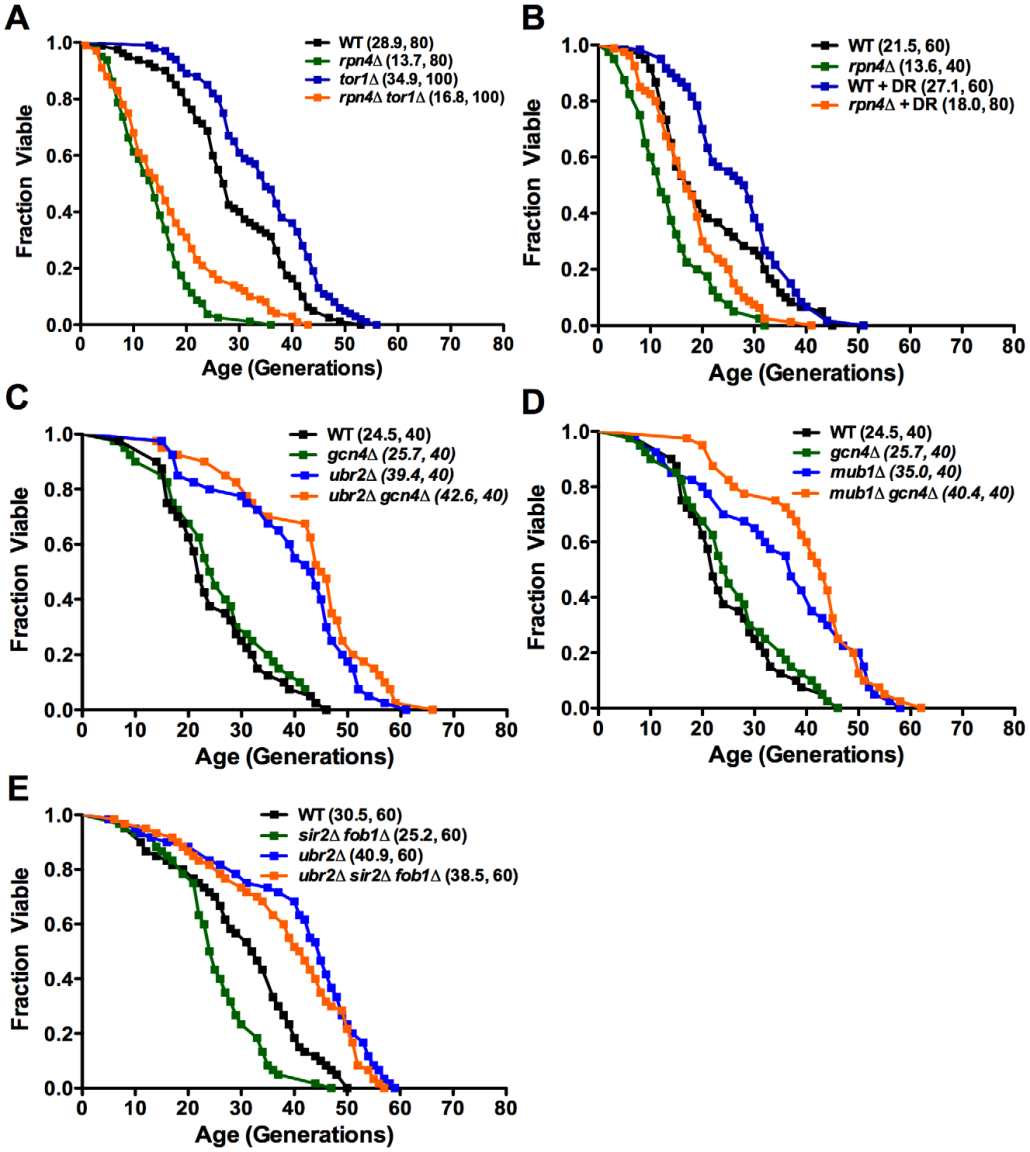
Impact of proteasome capacity on longevity is largely unaffected by known lifespan mediating pathways. Mean lifespan and cell counts are shown in parenthesis. (A) *rpn4Δ* induced lifespan shortening is partially rescued by *tor1Δ* or (B) by dietary restriction (DR). Survival curves of *rpn4Δ*, *tor1Δ* and *rpn4Δ tor1Δ* or WT and *rpn4Δ *in the presence of 2% (A) or 0.05% glucose (B) (DR). (C) *ubr2Δ* and (D) *mub1Δ* mediated lifespan extension is independent of *GCN4*. Survival curves of *gcn4Δ, ubr2Δ*, and *ubr2Δ gcn4Δ* or *gcn4Δ, mub1Δ*, and *mub1Δ gcn4Δ* cells. (E) *ubr2Δ* mediated lifespan extension is independent of *SIR2 and FOB1*. Survival curves of *sir2Δ fob1Δ, ubr2Δ*, and *ubr2Δ sir2Δ fob1Δ *cells. A statistical analysis of the data is summarized in Table S2.

Previously, aging pathways have been linked to mitigation of proteotoxic stress. For instance, dietary restriction and reduced TOR signaling have been suggested to extend lifespan, at least in part, through effects on mRNA translation and enhanced autophagy in yeast, nematodes, and fruit flies [42]. Deletion of *TOR1*, which codes for one of two partially redundant TOR kinases, has been previously shown to extend RLS, and inhibition of Tor1 is thought to mediate RLS extension from dietary restriction [29]. Our findings indicate that deletion of *TOR1* extends the lifespan of *rpn4Δ* mother cells (Figure 9A). Likewise, dietary restriction by reducing the glucose concentration of the medium also increased the lifespan of cells lacking *RPN4* (Figure 9B). Deletion of *GCN4*, which has been previously shown to be required for full lifespan extension in response to dietary restriction or deletion of *TOR1* [30], does not affect full lifespan extension of *ubr2Δ* or *mub1Δ* cells (Figure 9C, 9D). These data support the conclusion that the effect of the proteasome on longevity pathways is at least partially distinct from dietary restriction/TOR signaling. Finally, *ubr2Δ* cells retain the ability to enhance longevity in a strain lacking both *SIR2* and *FOB1* (Figure 9E), indicating that increased Sir2 activity also does not account for lifespan extension by elevated proteasome function. These findings are consistent with the model that enhanced proteasome activity increases RLS through a mechanism at least partially distinct from the major known longevity pathways in yeast.

## Discussion

In this report we took advantage of the tight transcriptional regulation of UPS components to increase the activity of the ubiquitin/proteasome system. Through inhibiting the regulated turnover of the proteasome-related transcription factor Rpn4 we were able to increase proteasome abundance and activity ∼1.5–2 fold. This strategy allowed us to investigate the effect of improved proteasome capacity on yeast RLS. Our data demonstrate that proteasome abundance and activity correlates with lifespan; enhanced proteasome capacity dramatically increases longevity in yeast, conferring an approximately 70% increase of medium and maximum RLS. This is a robust effect, comparable to the longest-lived single gene deletions identified and greater than the extension observed either by deletion of *TOR1* or overexpression of *SIR2* [29]–[31], [43]. Given the high conservation of the proteasome in all eukaryotes and a host of literature on the role of declining proteasome function in aging cells and tissues in different organisms [8]–[14], we speculate that increased proteasome function might also positively affect aging in higher eukaryotes. This hypothesis is supported by studies, which revealed elevated proteasome capacity in long-lived organisms, such as centenarians or naked mole rats [13], [14].

Although the precise mechanism is yet to be elucidated, our results suggest that improved protein quality control, and thus protein homeostasis, might be the origin of extended lifespan in cells with increased proteasome capacity. Cells with elevated proteasome capacity retain rapid growth under protein stress inducing conditions and are able to turnover structurally unstable and aggregation-prone proteins at an enhanced rate. We cannot rule out the possibility that increased proteasome capacity also enhances turnover of negative regulators of lifespan in *S. cerevisiae*. However, we have no evidence supporting this hypothesis. Interestingly, the effect of the proteasome on lifespan appears to be at least partially independent of known longevity-modulating pathways in yeast, a phenomenon which might be explained by the relatively autonomous regulatory mechanism for proteasome level adaptation in response to proteotoxic stress, as described above. Yet cross-communication between the proteasome and TORC1 cannot be excluded at this point, since the full lifespan extension from *TOR1* deletion was not achieved in cells with reduced proteasome capacity. In this regard, it is worth noting that transcription of *RPN4* is controlled by *HSF1*, whose worm counterpart is required for lifespan extension by dietary restriction [44].

Similar to proteasome function, autophagy and the proteolytic capacity of the lysosome declines with age in model organisms and in mammals [45]. Many interventions, such as dietary restriction, drug treatment, overexpression of sirtuins [46] or restoration of autophagosomal function in old animals via transgenes in mice or flies [47], [48], result in increased autophagy and lifespan extension. In light of the results reported here, we conclude that both proteolytic systems appear to play important roles in determining longevity. However, autophagy is also involved in apoptotic cell death and may participate in limiting lifespan as suggested by reports in *C. elegans* [49]. Thus, approaches to increase UPS function in aging cells might represent viable alternative strategies for modulating longevity and for future anti-aging drug development.

Intriguingly, the autophagic and proteasomal systems also communicate [50]. Inhibition of the proteasome induces autophagy, and in higher eukaryotes the lysosome is able to internalize ubiquitinated proteins via specific ubiquitin receptors involved in autophagosome formation [50]. Despite this, enhanced autophagy is clearly unable to fully compensate for impaired proteasome function with respect to yeast longevity, since we find that proteasome defects lead to short lifespan. A major challenge for the future will be to determine the detailed mechanisms by which these proteolytic pathways modulate longevity and what role they play during mammalian aging.

## Materials and Methods

### Strains and plasmids

The strains used in this study are listed in Table S1. All strains are isogenic to BY4741 or BY4742 [51] and are S288C-derived, except the strains used in Figure 1A. DY62, DY93, DY100, DY106, DY155 and their parental strain SUB62 were kindly provided by Daniel Finley. Complete genomic gene deletion was achieved by homologous recombination using standard techniques [52], [53]. The plasmids pYES25Q-CFP and pYES103Q-CFP were a kind gift of Michael Sherman and the plasmid pRP44 was kindly provided by Davis Ng.

### Replicative lifespan analysis

Replicative lifespan assays were carried out as described previously [54]. Unless otherwise noted, all lifespan experiments were performed on YPD plates with 2% glucose. Lifespan assays under dietary restriction (DR) were performed on YPD with 0.05% glucose. Lifespan curves in Figure 1A–1D, Figure 4A, and Figure 9A, 9B, 9E were compiled from multiple assays containing experimentally matched WT and single mutant controls. The strains used for Figure 1B were congenic.

### Quantitative real-time PCR analysis

Strains were grown to mid-log phase (O.D. _600nm_ = 0.5). Cell extracts were prepared with glass bead lysis. Total RNA was purified using a QIAGEN RNeasy kit according to the manufacturer's instructions. 1 µg RNA was reverse transcribed using Bio-Rad's iScript cDNA synthesis kit containing oligo(dT) and random hexamer primers. Lysate preparation, RNA purification and reverse transcription were performed on multiple biological samples in parallel. cDNA products were amplified with a Bio-Rad iCycler using SYBRGreen for detection according to manufacturer's recommendations. Primer sequences are listed in Table S7. Serial dilutions of *ubr2Δ* cDNA were used as standards. Six (Figure 4B) or nine (Figure 2B) technical replicates were performed from each of three biological samples for all strains and transcripts queried. The ratio of target to *PRP8* transcript signal was taken for experiment-matched technical replicates and then averaged for each biological sample. One-way ANOVA tests were performed between strain transcript level averages to assess significance using GraphPad's Prism software.

### Gel electrophoresis and immunoblotting

Anti-proteasome antibodies were obtained from BioMol (anti-CP) or a kind gift of Daniel Finley (anti-Rpn5 and anti-Rpn8) and anti-Pgk1 was from Invitrogen. Analysis of intact proteasome species in unfractionated lysate was done as described previously [55]. Briefly, post-diauxic shift cells were harvested, resuspended in lysis buffer (50 mM Tris-HCl, pH 7.5, 0.5 mM EDTA, 5 mM MgCl_2_, protease inhibitor cocktail tablet, Roche) and drop-frozen in liquid nitrogen. Frozen yeast cells were lysed by cryolysis using an MM301 grinding mill (Retsch, Germany) following the manufacturer protocol. Cell extracts were cleared at 13,000 rpm for 30 min at 4°C to remove insoluble material. Protein concentration was determined by a Bradford assay (BioRad) using bovine serum albumin as standard. After clarification of the lysate by centrifugation, protein content was assessed by a Bradford assay. Equal amount of total protein (150 µg Figure 2C, left panel, 200 µg Figure 2C, right panel) were applied to native gels composed of 3.5% acrylamide, 90 mM Tris-base, 90 mM boric acid, 1 mM ATP, 5 mM MgCl_2_, 0.5 mM EDTA, which were run in the same buffer omitting the acrylamide. The gels were run at 100V for 3 h at 10°C, incubated for 30 min at 30°C in substrate buffer (50 mM Tris-HCl [pH 7.4], 5 mM MgCl_2_, 1 mM ATP, 0.05 µM Suc-LLVY-AMC (Bachem) for the in-gel activity assay unless otherwise indicated. Active bands were visualized using an UV screen at 340 nm. To assess equal loading the same samples were subjected to SDS-PAGE followed by immunodetection with anti-CP and anti-Pgk1 antibodies.

### Proteasome activity assay

Post-diauxic shift cells were harvested, resuspended in lysis buffer (50 mM Tris-HCl, pH 7.5, 0.5 mM EDTA, 5 mM MgCl_2_, protease inhibitor cocktail tablet, Roche) and drop-frozen in liquid nitrogen. Frozen yeast cells were lysed by cryolysis using an MM301 grinding mill (Retsch, Germany) following the manufacturer protocol. Cell extracts were cleared at 13,000 rpm for 30 min at 4°C to remove insoluble material. Protein concentration was determined by a Bradford assay (BioRad) using bovine serum albumin as standard. Proteasomal chymotrypsin-like activity was assessed using Suc-LLVY-AMC (Bachem), Boc-RLR-AMC (Bachem) or Ac-nLPnL-AMC (Bachem). The assays were performed in lysis buffer in a final reaction volume of 200 µl with 50 µg total lysate protein in 96-well black microtitre plates (Costar) with 100 µM peptide substrate. The initial rate of fluorescence increase was recorded after incubation for 15 min at 30°C with an excitation wavelength of 380 nm and an emission wavelength of 460 nm in a SpectraMAX M5 multifunctional plate reader (Molecular Devices). The initial rates of peptide hydrolysis were determined in triplicates each in the presence and absence of the proteasome inhibitor MG132 (50 µM, Calbiochem).

### Growth curves

Cultures were inoculated for 36 h in YPD. Subsequently, 2.5 µl aliquots were added to 147.5 µl YPD in a HC2 plate. Growth was recorded in a Bioscreen C MB machine (Growth Curves USA) for 24 h at 30°C under continuous shaking and with 420–580 nm absorbance readings every 30 minutes.

### Selective reaction monitoring (SRM) of Rpn4 expression in total cell lysate

Yeast cells were grown in YPD to O.D._600nm_ 3. *RPN4* expression was induced by addition of 50 µM CdCl_2_ for 1 h at 30°C. Protein extracts were prepared using glass bead lysis in a buffer containing 6 M Urea/2 M ThioUrea/20 mM HEPES, pH 7.2. Peptides were generated using a two-step digest as described in deGodoy et al. [56] and separated on a 15 cm reverse phase column (3 µM Reprosil, Dr. Maisch, packed in house) using a 10 to 50% acetonitrile gradient. Peptides were sprayed directly into the mass spectrometer (4000 Q-TRAP, ABSciex) and ionized using a NanoII ion source (ABSciex). Data were recorded using the selected reaction-monitoring mode (SRM/MRM) of the instrument analyzed using the MultiQuant 1.3 (ABSciex) and R software packages (www.R-project.org) [57]. The different runs were normalized to the trace of the y12 fragment ion of an actin peptide (SYELPDGQVITIGNER).

### SILAC (stable isotope labeling in cell culture)–based proteomic comparison of *rpn4Δ* and *ubr2Δ*cells

Yeast strains (WT, *rpn4*Δ, *ubr2*Δ) were grown in complete minimal media supplemented with isotopically labeled lysine to O.D. _600nm_ 3. 50 µM CdCl_2_ was added and the cells were grown for an additional hour. Wild type cells were labeled with light (lysine 0), *rpn4*Δ cells with medium (lysine 4) and *ubr2*Δ cells with heavy lysine (lysine 8). Equal amounts of cells were mixed, resuspended in 1x SDS sample buffer and lysed using glass beads. After centrifugation the clear supernatant was concentrated in a Milipore Amicon ultra cartridge (10 kD cut-off). Proteins were separated on a 12% SDS-PAGE. The gel was cut in 12 slices and each slice was subjected to an in-gel digest using Endopeptidase LysC (Wako Chemicals) [56]. The recovered peptides were separated on a 15 cm reverse phase column (in house packed, 3 µm ReproSil-Pur C18, Dr. Maisch) and sprayed directly into the LTQ-OrbiTRAP mass spectrometer (Thermo). The recorded data were analyzed using the MaxQuant [58] and R software package (www.R-project.org).

### Phenotypic analysis of gene deletions

Overnight cultures were diluted to O.D. _660nm_ 0.1, incubated for 4 h at 30°C to obtain exponentially growing cultures. The cultures were diluted in 96 well plates with YPD to a density of 6×10^6^ cells per well, followed by 5-fold serial dilutions and spotted onto YPD plates in the absence or presence of 0.5 µg/ml tunicamycin (Sigma Aldrich), 50 µM cadmium chloride (Sigma Aldrich) or onto synthetic arginine drop out plates in the absence or presence of 3 µg/ml canavanine (Sigma Aldrich).

### Turnover of the unstable proteasome model substrate Δ2GFP

Overnight cultures of cells constitutively expressing Δ2GFP-HA were diluted to O.D. _660nm_ 0.5 and incubated for 2 h in synthetic media. New synthesis was blocked by the addition of 200 µg/ml CHX. At the time indicated aliquots were harvested and frozen in liquid nitrogen. After alkaline lysis [59] and measuring the total protein concentration with a Bradford reagent (Pierce), 20 µg were subjected to SDS-PAGE and immuno-detection using an anti-GFP polyclonal antibody (Clontech). Signals were visualized using ECL chemiluminescence in an ImageQuant LAS 4000 imager (GE Healthcare) and quantified using the ImageQuant software package.

### Visualization of PolyQ extended Huntingtin exon1 aggregation via live cell fluorescence microscopy

Cells were grown at 30°C on solid selective synthetic complete medium with 2% glucose. For induction of 25Q or 103Q, cells were transferred into liquid selective synthetic complete medium with 2% raffinose, induced by addition of 2% galactose and grown for the times indicated. For imaging, 1 O.D. _600nm_ of cells were transferred to ConA coated 35 mm imaging dishes, washed 3x and mounted to the microscope. CFP fluorescence images were taken using the Olympus BX81 microscope equipped with a 150x objective, a narrow band CFP filter and an Andor iXon8 EMCCD for data acquisition. All images were taken under the same conditions with 20–31 serial Z-sections (0.2 mm/section and 0.2 s exposure/section) to cover the thickness of the cells. For data acquisition the Andor imaging software IQ 2.2 were used. Z-stacks were merged with ImageJ 1.37v.

## Supporting Information

Figure S1 Pairwise *p*-value comparison of the qRT-PCR data presented in Figure 2B. Statistical significance was assessed by a one-way Anova analysis using the GraphPad Prism software.
(TIF)

Figure S2 Proteasomal peptidase activity in unfractionated lysates in the presence of the proteasome inhibitor MG132. The same samples as in Figure 3A were subjected to an analysis of the three distinct proteasomal activities in the presence of the proteasome-specific inhibitor MG132. The same scale is used as in Figure 3A. Note: MG132 only weakly affects the trypsin-like activity of the proteasome.
(TIF)

Figure S3 Average strain generation time calculated from the growth curves presented in Figure 3B.
(TIF)

Table S1 Strains used in this study.
(PDF)

Table S2 Statistical analysis of the RLS experiments presented. Curve comparisons were assessed by a Wilcoxon test. Rank sum p-values: * p < 0.05, ** p < 0.01, *** p < 0.001, ns  =  not significant.
(PDF)

Table S3 Proteins upregulated in *rpn4Δ* cells with a log2(ratio) > 0.5 relative to WT abundance after cadmium chloride treatment.
(PDF)

Table S4 Proteins downregulated in *rpn4Δ* cells with a log2(ratio) <−0.5 relative to WT abundance chloride treatment.
(PDF)

Table S5 Proteins upregulated in *ubr2Δ* cells with a log2(ratio) > 0.5 relative to WT abundance chloride treatment.
(PDF)

Table S6 Proteins downregulated in *ubr2Δ* cells with a log2(ratio) <−0.5 relative to WT abundance chloride treatment.
(PDF)

Table S7 Primer Sequences. This table lists the primer sequences used for qRT-PCR experiments.
(PDF)
