# Supplementary figures and images for: Elevated Proteasome Capacity Extends Replicative Lifespan in Saccharomyces cerevisiae
Source: PLoS Genet. 2011 Sep 8;7(9):e1002253. doi: 10.1371/journal.pgen.1002253 (PMC3169524; doi:10.1371/journal.pgen.1002253)

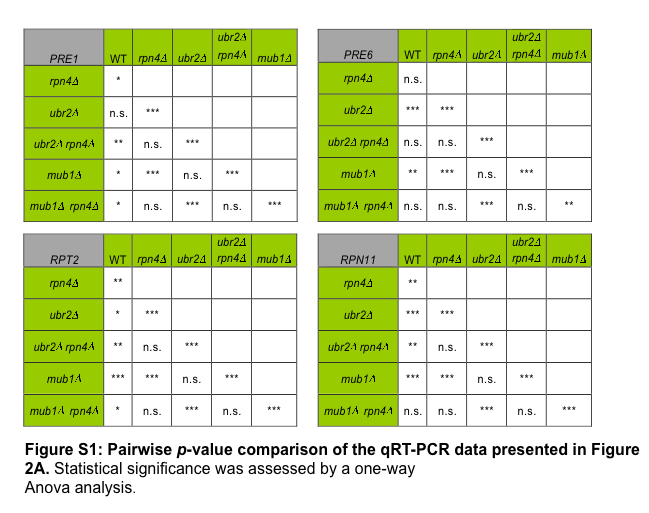

Supplement: Figure S1 — Pairwise p-value comparison of the qRT-PCR data presented in Figure 2B. Statistical significance was assessed by a one-way Anova analysis using the GraphPad Prism software. (TIF) [file pgen.1002253.s001.tif]

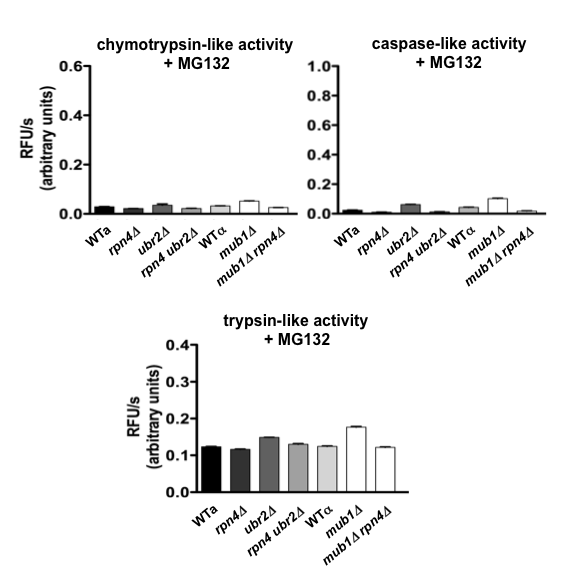

Supplement: Figure S2 — Proteasomal peptidase activity in unfractionated lysates in the presence of the proteasome inhibitor MG132. The same samples as in Figure 3A were subjected to an analysis of the three distinct proteasomal activities in the presence of the proteasome-specific inhibitor MG132. The same scale is used as in Figure 3A. Note: MG132 only weakly affects the trypsin-like activity of the proteasome. (TIF) [file pgen.1002253.s002.tif]

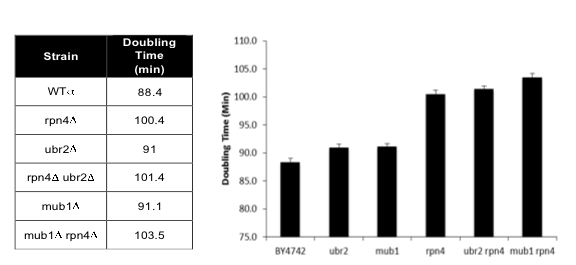

Supplement: Figure S3 — Average strain generation time calculated from the growth curves presented in Figure 3B. (TIF) [file pgen.1002253.s003.tif]
