# Supplementary material for: Elevated Proteasome Capacity Extends Replicative Lifespan in Saccharomyces cerevisiae
Source: PLoS Genet. 2011 Sep 8;7(9):e1002253. doi: 10.1371/journal.pgen.1002253 (PMC3169524; doi:10.1371/journal.pgen.1002253)
Supplement: Table S1 — Strains used in this study. (PDF) [file pgen.1002253.s004.pdf]

**Table S1: Strains used in this study**

| Strains | Genotype | Figure | Ref. |
|---|---|---|---|
| SUB62 | MAT**a** *lys2-801 leu2-3, 2-112 ura3-52 his3-Δ200 trp1-1* | 1A | [23] |
| DY106 | MAT**a** *lys2-801 leu2-3 2-112 ura3-52 his3-Δ200 trp1-1(am) rpt1K256S* | 1A | [23] |
| DY62 | MAT**a** *lys2-801 leu2-3 2-112 ura3-52 his3-Δ200 trp1-1(am) rpt2K229R S241F* | 1A | [23] |
| DY93 | MAT**a** *lys2-801 leu2-3 2-112 ura3-52 his3-Δ200 trp1-1(am) rpt3K219R* | 1A | [23] |
| DY155 | MAT**a** *lys2-801 leu2-3 2-112 ura3-52 his3-Δ200 trp1-1(am) rpt5K228R* | 1A | [23] |
| DY100 | MAT**a** *lys2-801 leu2-3 2-112 ura3-52 his3-Δ200 trp1-1(am) rpt6K195R* | 1A | [23] |
| LF960 | MATα *his3Δ1 leu2Δ0 lys2Δ0 ura3Δ0* | 1C, E, 2B-E, 9A-E | [60] |
| DH461 | Matα *his3Δ1 leu2Δ0 lys2Δ0 tor1Δ::URA3* | 9A | [29] |
| ND93 | Matα *his3Δ1 lys2Δ0 ura3Δ0 sir2Δ::HIS3 fob1Δ::LEU2* | 9E | [61] |
| LF1030 | MATα *his3Δ1 leu2Δ0 lys2Δ0 ura3Δ0 gcn4Δ::KanMX* | 9C, D | [30] |
| GS1821 | MAT**a** *his3Δ1 leu2Δ0 met15Δ0 ura3Δ0* | 4A | [60] |
| KS2 | MAT**a** *his3Δ1 leu2Δ0 met15Δ0 ura3Δ0* | 2C-E, 3, 5, 6 | [60] |
| GS1875 | MATα *his3Δ1 leu2Δ0 lys2Δ0 ura3Δ0* | 1B, D | [60] |
| yMS153 | MATα *his3Δ1 leu2Δ0 met15Δ0 ura3Δ0 rpn4Δ::KanMX* | 1B | this study |
| yMS268 | MATα *his3Δ1 leu2Δ0 met15Δ0 ura3Δ0* | 1B | this study |
| yMS485 | MAT**a** *his3Δ1 leu2Δ0 met15Δ0 ubp6Δ::URA3* | 1B | this study |
| yMS822 | MAT**a** *his3Δ1 leu2Δ0 lys2Δ0 ura3Δ0 rpn4Δ::HphMX* | 1C, 2C-E, 3, 5, 6 | this study |
| yMS831 | MAT**a** *his3Δ1 leu2Δ0 met15Δ0 ura3Δ0* | 1C, 3, 4A, B, 5, 6 | [60] |
| yMS921 | MAT**a** *his3Δ1 leu2Δ0 met15Δ0 ura3Δ0 pre9Δ::KanMX* | 1B, 1F | [60] |
| yMS973 | MAT**a** *his3Δ1 leu2Δ0 met15Δ0 ura3Δ0 ump1Δ::HphMX* | 1B | this study |
| yMS1022 | MAT**a** *his3Δ1 leu2Δ0 met15Δ0 ura3Δ0 ire1Δ::KanMX* | 6B | [60] |
| yMS1148 | MAT**a** *his3Δ1 leu2Δ0 lys2Δ0 ura3Δ0 ubr2Δ::NatMX* | 1C, 1F, 2C-E, 3, 4A, 5, 6 | this study |
| yMS1149 | MAT**a** *his3Δ1 leu2Δ0 lys2Δ0 ura3Δ0 rpn4Δ::HphMX ubr2Δ::NatMX* | 1C, 2C-E, 3, 4A, 5, 6 | this study |
| yMS1184 | MATα *his3Δ1 leu2Δ0 lys2Δ0 ura3Δ0 ump1Δ::HphMX* | 1B | this study |
| yMS1371 | MAT**a** *his3Δ1 leu2Δ0 met15Δ0 ura3Δ0 + 25Q-CFP-pYES2* | 8C | this study |
| yMS1373 | MAT**a** *his3Δ1 leu2Δ0 lys2Δ0 ura3Δ0 ubr2Δ::NatMX + 25Q-CFP-pYES2* | 8C | this study |
| yMS1377 | MAT**a** *his3Δ1 leu2Δ0 met15Δ0 ura3Δ0 + 103Q-CFP-pYES2* | 8A | this study |
| yMS1379 | MAT**a** *his3Δ1 leu2Δ0 lys2Δ0 ura3Δ0 ubr2Δ::NatMX + 103Q-CFP-pYES2* | 8B | this study |

| | | | |
|---|---|---|---|
| yMS1408 | MAT**a** *his3Δ1 leu2Δ0 lys2Δ0 ura3Δ0 + pRP44* | 7 | this study |
| yMS1409 | MAT**a** *his3Δ1 leu2Δ0 lys2Δ0 ura3Δ0 rpn4Δ::HphMX + pRP44* | 7 | this study |
| yMS1410 | MAT**a** *his3Δ1 leu2Δ0 met15Δ0 ura3Δ0 ubr2Δ::NatMX + pRP44* | 7 | this study |
| yMS1411 | MAT**a** *his3Δ1 leu2Δ0 lys2Δ0 ura3Δ0 rpn4Δ::HphMX ubr2Δ::NatMX + pRP44* | 7 | this study |
| yMS1442 | MAT**a** *his3Δ1 leu2Δ0 met15Δ0 ura3Δ0 yap1Δ*::KanMX | 4A | [60] |
| yMS1446 | MAT**a** *his3Δ1 leu2Δ0 met15Δ0 ura3Δ0 ubr2Δ::NatMX yap1Δ::KanMX* | 4A | this study |
| yMS1473 | MAT**a** *his3Δ1 leu2Δ0 met15Δ0 ura3Δ0 pre9Δ::KanMX ubr2Δ::NatMX* | 1F | this study |
| BR2056 | MATα *his3Δ1 leu2Δ0 lys2Δ0 ubr2Δ::URA3* | 9C, E | this study |
| BR2170 | MATα *his3Δ1 lys2Δ0 ubr2Δ::URA3 fob1Δ::KanMX sir2::LEU2* | 9E | this study |
| BR2194 | MATα *his3Δ1 leu2Δ0 met15Δ0 ubr2Δ::URA3* | 1F | this study |
| BR2200 | MAT**a** *his3Δ1 leu2Δ0 met15Δ0 ura3Δ0* | 1D, F, 4A | [60] |
| BR2203 | MATα *his3Δ1 leu2Δ0 lys2Δ0 ura3Δ0 ubr2Δ::KanMX rpn4Δ::KanMX* | 1C, 2B | this study |
| BR2206 | MATα *his3Δ1 leu2Δ0 lys2Δ0 ura3Δ0 rpn4Δ::KanMX tom1Δ::KanMX* | 1E | this study |
| BR2264 | MATα *his3Δ1 leu2Δ0 lys2Δ0 ura3Δ0 mub1Δ::KanMX rpn4Δ::KanMX* | 1D, 2B | this study |
| BR2276 | MATα *his3Δ1 leu2Δ0 lys2Δ0 gcn4Δ::KanMX ubr2Δ::URA3* | 9C | this study |
| BR2287 | MATα *his3Δ1 leu2Δ0 lys2Δ0 tor1Δ::URA3 rpn4Δ::KanMX* | 9A | this study |
| BR2296 | MATα *his3Δ1 leu2Δ0 lys2Δ0 ura3Δ0 gcn4Δ::KanMX mub1Δ::KanMX* | 9D | this study |
| BR2306 | MATα *his3Δ1 lys2Δ0 ura3Δ0 mub1Δ::LEU2* | 1D | this study |
| BR2307 | MATα *his3Δ1 lys2Δ0 ura3Δ0 mub1Δ::LEU2* | 1D | this study |
| BR2308 | MAT**a** *his3Δ1 leu2Δ0 met15Δ0 ura3Δ0 mub1::LEU2* | 4A | this study |
| BR2309 | MAT**a** *his3Δ1 leu2Δ0 met15Δ0 ura3Δ0 mub1::LEU2* | 4A | this study |
| BR2322 | MATα *his3Δ1 leu2Δ0 lys2Δ0 ura3Δ0 rpn4Δ::KanMX* | 1C, D, 2B, 9B | [60] |
| BR2324 | MATα *his3Δ1 leu2Δ0 lys2Δ0 ura3Δ0 tom1Δ::KanMX* | 1E | [60] |
| BR2325 | MATα *his3Δ1 leu2Δ0 lys2Δ0 ura3Δ0 mub1Δ::KanMX* | 1D, 2B-E, 3, 5, 6, 9D | [60] |
| BR2326 | MAT**a** *his3Δ1 leu2Δ0 met15Δ0 ura3Δ0 mub1Δ::KanMX* | 4A | [60] |
| BR2327 | MATα *his3Δ1 leu2Δ0 lys2Δ0 ura3Δ0* ubr2::KanMX | 2B, | [60] |
| BR2403 | MAT**a** *his3Δ1 leu2Δ0 ura3Δ0 ubr2Δ::KanMX yap1Δ*::KanMX | 4A | this study |
| BR2415 | MAT**a** *his3Δ1 leu2Δ0 met15Δ0 ura3Δ0 mub1Δ::KanMX yap1Δ*::KanMX | 4A | this study |
