# Supplementary material for: Elevated Proteasome Capacity Extends Replicative Lifespan in Saccharomyces cerevisiae
Source: PLoS Genet. 2011 Sep 8;7(9):e1002253. doi: 10.1371/journal.pgen.1002253 (PMC3169524; doi:10.1371/journal.pgen.1002253)
Supplement: Table S2 — Statistical analysis of the RLS experiments presented. Curve comparisons were assessed by a Wilcoxon test. Rank sum p-values: * p < 0.05, ** p < 0.01, *** p < 0.001, ns = not significant. (PDF) [file pgen.1002253.s005.pdf]

**Table S2: Statistical analysis of the RLS experiments presented**.
*P*-value matrices for Figure 1, 4A and 9. *P*-values were assessed by a Wilcoxon test.
Ranksum *p*-values: * $p < 0.05$, ** $p < 0.01$, *** $p < 0.001$, n.s. = not significant

| Figure 1A | WT SUB62 | *rpt1S* | *rpt2RF* | *rpt3R* | *rpt5R* |
|---|---|---|---|---|---|
| *rpt1S* | ** | | | | |
| *rpt2RF* | *** | *** | | | |
| *rpt3R* | *** | ** | ** | | |
| *rpt5R* | *** | *** | *** | *** | |
| *rpt6R* | *** | *** | n.s. | * | *** |

| Figure 1B | WT | *rpn4Δ* | *ump1Δ* | *ubp6Δ* |
|---|---|---|---|---|
| *rpn4Δ* | *** | | | |
| *ump1Δ* | *** | n.s. | | |
| *ubp6Δ* | *** | * | n.s. | |
| *pre9Δ* | *** | *** | ** | * |

| Figure 1C | WT | *rpn4Δ* | *ubr2Δ* |
|---|---|---|---|
| *rpn4Δ* | *** | | |
| *ubr2Δ* | *** | *** | |
| *ubr2Δ rpn4Δ* | *** | n.s. | *** |

| Figure 1D | WT | *rpn4Δ* | *mub1Δ* |
|---|---|---|---|
| *rpn4Δ* | *** | | |
| *mub1Δ* | *** | *** | |
| *mub1Δ rpn4Δ* | *** | n.s. | *** |

| Figure 1E | WT | *rpn4Δ* | *tom1Δ* |
|---|---|---|---|
| *rpn4Δ* | *** | | |
| *tom1Δ* | ** | *** | |
| *tom1Δ rpn4Δ* | n.s. | *** | *** |

| Figure 1F | WT | *pre9Δ* | *ubr2Δ* |
|---|---|---|---|
| *pre9Δ* | *** | | |
| *ubr2Δ* | *** | *** | |
| *ubr2Δ pre9Δ* | *** | n.s. | *** |

| Figure 4A (L) | WT | *yap1Δ* | *ubr2Δ* |
|---|---|---|---|
| *yap1Δ* | *** | | |
| *ubr2Δ* | *** | *** | |
| *ubr2Δ yap1Δ* | n.s. | *** | *** |

| Figure 4A (R) | WT | *yap1Δ* | *mub1Δ* |
|---|---|---|---|
| *yap1Δ* | *** | | |
| *mub1Δ* | *** | *** | |
| *mub1Δ yap1Δ* | ** | *** | *** |

| Figure 9A | WT | *rpn4Δ* | *tor1Δ* |
|---|---|---|---|
| *rpn4Δ* | *** | | |
| *tor1Δ* | ** | *** | |
| *rpn4Δ tor1Δ* | *** | * | *** |

| Figure 9B | WT | *rpn4Δ* | WT + DR |
|---|---|---|---|
| *rpn4Δ* | *** | | |
| WT + DR | ** | *** | |
| *rpn4Δ* + DR | n.s. | ** | *** |

| Figure 9C | WT | *gcn4Δ* | *ubr2Δ* |
|---|---|---|---|
| *gcn4Δ* | ns | | |
| *ubr2Δ* | *** | *** | |
| *ubr2Δ gcn4Δ* | *** | *** | n.s. |

| Figure 9D | WT | *gcn4Δ* | *mub1Δ* |
|---|---|---|---|
| *gcn4Δ* | *** | | |
| *mub1Δ* | *** | ** | |
| *mub1Δ gcn4Δ* | *** | *** | n.s. |

| Figure 9E | WT | *sir2Δ fob1Δ* | *ubr2Δ* |
|---|---|---|---|
| *sir2Δ fob1Δ* | ** | | |
| *ubr2Δ* | *** | *** | |
| *ubr2Δ sir2Δ fob1Δ* | *** | *** | n.s. |
