# Supplementary material for: Elevated Proteasome Capacity Extends Replicative Lifespan in Saccharomyces cerevisiae
Source: PLoS Genet. 2011 Sep 8;7(9):e1002253. doi: 10.1371/journal.pgen.1002253 (PMC3169524; doi:10.1371/journal.pgen.1002253)
Supplement: Table S3 — Proteins upregulated in rpn4Δ cells with a log2(ratio) > 0.5 relative to WT abundance after cadmium chloride treatment. (PDF) [file pgen.1002253.s006.pdf]

Table S3: Proteins with log2 >0.5 fold increased abundance in *rpn4Δ* cells relative to WT.

1	COX1_YEAST;Q36738_YEAST;Q36739_YEAST;Q36740_YEAST;Q95947_YEAST
2	RMAR_YEAST
3	GPR1_YEAST;YJF5_YEAST
4	CYS3_YEAST
5	CCR4_YEAST
6	PMT2_YEAST
7	MYO4_YEAST
8	KPYK1_YEAST
9	PTA1_YEAST
10	AIM2_YEAST
11	ACS1_YEAST;Q66RJ0_YEAST
12	CALX_YEAST
13	BDH1_YEAST;Q6B208_YEAST
14	PUR7_YEAST
15	CACM_YEAST
16	OSH1_YEAST
17	IMDH2_YEAST;IMDH3_YEAST;IMDH1_YEAST
18	SLA1_YEAST
19	ACH1_YEAST
20	PEP1_YEAST
21	LONM_YEAST
22	RL19_YEAST
23	YB029_YEAST
24	STU1_YEAST
25	AP2A_YEAST
26	QCR1_YEAST
27	PIN4_YEAST
28	YBF5_YEAST;Q6B2V4_YEAST
29	PRX1_YEAST
30	RS8_YEAST
31	SYIC_YEAST
32	NU170_YEAST
33	YBI6_YEAST;Q6B2T6_YEAST
34	RL23_YEAST
35	ATM_YEAST
36	AMPM2_YEAST
37	RL32_YEAST
38	CND2_YEAST
39	O13449_YEAST;ATPA_YEAST
40	YBK4_YEAST
41	O42833_YEAST;UGA2_YEAST
42	TTP1_YEAST
43	IMB2_YEAST
44	ATPG_YEAST
45	QOR_YEAST
46	RS11_YEAST
47	YBQ6_YEAST
48	HSP26_YEAST
49	YBS4_YEAST
50	ECM33_YEAST
51	EIF3A_YEAST
52	IST2_YEAST
53	PPA5_YEAST;Q06856_YEAST;Q06876_YEAST

54	PHO88_YEAST
55	YBV8_YEAST
56	TKT2_YEAST
57	EF1A_YEAST
58	YBY9_YEAST
59	IRA1_YEAST
60	ERF1_YEAST
61	ARA1_YEAST
62	MKAR_YEAST
63	HSP79_YEAST
64	RS9A_YEAST;RS9B_YEAST
65	RL21A_YEAST;RL21B_YEAST
66	G6PI_YEAST
67	KTR4_YEAST
68	SDS24_YEAST
69	GLU2A_YEAST
70	OM14_YEAST
71	RGD1_YEAST
72	APE3_YEAST
73	SNF5_YEAST
74	SRO9_YEAST
75	PDI_YEAST
76	YCE5_YEAST
77	SPB1_YEAST
78	YCF7_YEAST
79	RV161_YEAST
80	ADP1_YEAST
81	CWH43_YEAST
82	NPP1_YEAST
83	RS14B_YEAST;RS14A_YEAST
84	THRC_YEAST;Q66RI3_YEAST
85	CTR86_YEAST
86	PAT1_YEAST
87	TUP1_YEAST
88	NOT1_YEAST
89	FBRL_YEAST
90	TSC13_YEAST
91	OSH2_YEAST
92	GPD2_YEAST;GPD1_YEAST
93	ARP2_YEAST
94	DBP10_YEAST
95	NPC2_YEAST
96	PLSC_YEAST;Q6B238_YEAST
97	PBP4_YEAST
98	MPG1_YEAST
99	RL31A_YEAST;RL31B_YEAST;Q06739_YEAST
100	UBX3_YEAST
101	TRM3_YEAST
102	KAR_YEAST
103	CDC48_YEAST
104	HOSM_YEAST;Q6B1Y9_YEAST
105	CDC53_YEAST
106	GDIR_YEAST
107	RL35_YEAST
108	ARF2_YEAST;ARF1_YEAST

109 RPN5_YEAST
110 DHH1_YEAST
111 GLT1_YEAST
112 GLE1_YEAST
113 NHP2_YEAST
114 DHE2_YEAST
115 FMP45_YEAST
116 HBT1_YEAST
117 HSP75_YEAST;Q05833_YEAST;HSP76_YEAST
118 YPD1_YEAST
119 YRB1_YEAST
120 SNQ2_YEAST
121 GCST_YEAST
122 SYSC_YEAST
123 VPS54_YEAST
124 AROF_YEAST
125 SYKC_YEAST;Q9HE17_YEAST
126 TPIS_YEAST
127 PST1_YEAST
128 MAK21_YEAST
129 LCB2_YEAST
130 TPS2_YEAST
131 SC61G_YEAST
132 YH11B_YEAST;YP13B_YEAST;YD14B_YEAST;YD11B_YEAST;YD12B_YEAST;YB12B_YEAST;YE12B_YEAST;YL11B_YEAST;YG13B_YEAST;YM14B_YEAST;YO12B_YEAST;YM11B_YEAST;YH11A_YEAST;YB12A_YEAST;YD11A_YEAST;YD12A_YEAST;YD14A_YEAST;YO12A_YEAST;YM11A_YEAST
133 BMH2_YEAST;BMH1_YEAST
134 ARX1_YEAST
135 VBA4_YEAST
136 TRM1_YEAST;Q9URQ6_YEAST;Q9Y747_YEAST
137 KIN1_YEAST
138 ARO1_YEAST
139 TAF12_YEAST
140 ODO2_YEAST
141 ENT5_YEAST
142 CYPH_YEAST
143 DHAS_YEAST
144 SEC5_YEAST
145 SEC7_YEAST
146 SCC2_YEAST
147 TCPZ_YEAST
148 MS116_YEAST
149 AHA1_YEAST
150 GLU2B_YEAST
151 HSP78_YEAST
152 SGPL_YEAST
153 RSC3_YEAST
154 SSF1_YEAST;SSF2_YEAST
155 UBX5_YEAST
156 YD333_YEAST
157 MSN5_YEAST
158 HXT7_YEAST;HXT6_YEAST
159 HXT3_YEAST
160 SVF1_YEAST

161 GGA1_YEAST
162
YA11B_YEAST;YP12B_YEAST;YN12B_YEAST;YD15B_YEAST;YL14B_YEAST;TY1AB_YEAST;YL12B_YEAST;YE11B_YEAST;Q03970_YEAST;YP11B_YEAST;YP14B_YEAST;YD13B_YEAST;YA11A_YEAST;YD13A_YEAST;TY1A_YEAST;YD15A_YEAST;YO11B_YEAST;YL14A_YEAST;YP14A_YEAST;YN12A_YEAST;YO11A_YEAST
163 ARO10_YEAST
164 YRA1_YEAST
165 RLA4_YEAST
166 EF2_YEAST
167 RV167_YEAST
168 SAC7_YEAST
169 UBA2_YEAST
170 RPN9_YEAST
171 NOP3_YEAST
172 GPI17_YEAST
173 PPZ2_YEAST
174 UTP6_YEAST
175 UGO1_YEAST
176 PPB_YEAST;Q66RD0_YEAST
177 KRE2_YEAST
178 RIB3_YEAST
179 LCD1_YEAST
180 EUG1_YEAST;Q6B1W0_YEAST
181 YD539_YEAST
182 OSTB_YEAST
183 GLGB_YEAST
184 UCRI_YEAST
185 ATC6_YEAST
186 IF5A2_YEAST
187 DPO5_YEAST
188 AGM1_YEAST
189 PRTB_YEAST
190 RPN3_YEAST
191 SAHH_YEAST
192 ERG28_YEAST
193 CAJ1_YEAST
194 GIP2_YEAST
195 HIS1_YEAST
196 HMF1_YEAST
197 THO1_YEAST
198 YEP7_YEAST
199 ARG56_YEAST
200 RIR1_YEAST
201 ALDH5_YEAST
202 RS24_YEAST
203 YER0_YEAST
204 THDH_YEAST
205 TRPE_YEAST
206 METE_YEAST;Q27JJ6_YEAST;Q27JK0_YEAST
207 SCS2_YEAST
208 KC13_YEAST
209 GDI1_YEAST
210 TBP_YEAST
211 BEM2_YEAST

212 ATC5_YEAST
213 SEC4_YEAST
214 BLM10_YEAST
215 MDJ1_YEAST
216 DLDH_YEAST
217 CAF16_YEAST
218 RL22B_YEAST
219 YPT1_YEAST
220 A4URX6_YEAST;A4URX7_YEAST;A4URX8_YEAST;A4URX9_YEAST;A4URY0_YEAST;A4URY1_YEAST;A4URY2_YEAST;A4URY3_YEAST;A4URY4_YEAST;A4URY5_YEAST;ACT_YEAST;Q7Z9V1_YEAST
221 PMM_YEAST
222 EMP47_YEAST
223 NIC96_YEAST
224 YFI6_YEAST
225 CDC14_YEAST
226 RL2_YEAST
227 SA155_YEAST
228 PMA1_YEAST
229 LEUC_YEAST
230 PDR1_YEAST
231 STT3_YEAST
232 TRP_YEAST
233 MTC2_YEAST
234 PNC1_YEAST
235 HEM2_YEAST
236 IF4F2_YEAST
237 YGF9_YEAST
238 YBP2_YEAST
239 ALG2_YEAST
240 RL7A_YEAST
241 YGI2_YEAST;Q6Q536_YEAST
242 RL28_YEAST
243 G4P1_YEAST
244 RMD9_YEAST
245 SNF4_YEAST
246 ITC1_YEAST
247 YGO0_YEAST
248 HUL5_YEAST
249 RL9A_YEAST
250 MAN1_YEAST
251 YIP5_YEAST
252 ATC1_YEAST
253 XRN1_YEAST
254 STR3_YEAST
255 GCN1_YEAST
256 KEX1_YEAST
257 CLH_YEAST
258 SPT16_YEAST
259 YGX8_YEAST
260 SEC15_YEAST
261 CSE1_YEAST
262 HXKB_YEAST
263 T2FB_YEAST
264 NMA1_YEAST;NMA2_YEAST

265 RS25A_YEAST;RS25B_YEAST
266 RL26B_YEAST;RL26A_YEAST
267 TAL2_YEAST;Q45U40_YEAST;Q6Q5P8_YEAST
268 UFD1_YEAST
269 PUR4_YEAST;Q45U25_YEAST
270 EI2BD_YEAST
271 PIL1_YEAST
272 GYP2_YEAST
273 SPT6_YEAST
274 YG35_YEAST
275 RL24B_YEAST
276 O94073_YEAST;CBS_YEAST
277 PEM1_YEAST
278 NSR1_YEAST
279 IF4F1_YEAST
280 RIR4_YEAST
281 SYYC_YEAST
282 G3P3_YEAST
283 FYV8_YEAST
284 ZPR1_YEAST
285 XPO1_YEAST
286 HSV2_YEAST
287 AP3B_YEAST
288 SYMC_YEAST
289 YG5L_YEAST
290 SLH1_YEAST
291 RL14B_YEAST;RL14A_YEAST
292 YHC1_YEAST
293 ECM29_YEAST
294 RL8A_YEAST
295 YHD9_YEAST
296 GPA1_YEAST
297 SODM_YEAST
298 ARLY_YEAST
299 SYNC_YEAST
300 YHI0_YEAST
301 RS27B_YEAST;RS27A_YEAST
302 DAP2_YEAST;Q66R87_YEAST
303 PUT2_YEAST
304 NCPR_YEAST
305 YHK5_YEAST
306 AAP1_YEAST
307 FSH1_YEAST
308 YHL4_YEAST;RSC30_YEAST
309 PANE_YEAST
310 SSZ1_YEAST
311 YHO0_YEAST
312 KSP1_YEAST
313 SFB3_YEAST
314 GRE3_YEAST
315 CDC12_YEAST
316 GGA2_YEAST
317 LSM12_YEAST
318 EPT1_YEAST
319 SS100_YEAST

320	YHU6_YEAST
321	FMO1_YEAST
322	6PGD1_YEAST
323	KOG1_YEAST
324	ELP5_YEAST
325	GPI16_YEAST
326	FDFT_YEAST
327	RIX1_YEAST
328	BCA1_YEAST
329	YH09_YEAST;Q6Q5R3_YEAST
330	SCC3_YEAST
331	ACA2_YEAST
332	NCB5R_YEAST
333	MET30_YEAST
334	SYG1_YEAST
335	MMF1_YEAST
336	RL34B_YEAST;RL34A_YEAST
337	YRB2_YEAST
338	RPN2_YEAST
339	SYTC_YEAST
340	AVT7_YEAST
341	MOB1_YEAST
342	6P21_YEAST
343	YIK8_YEAST;Q6B2N8_YEAST
344	SEC24_YEAST
345	ODO1_YEAST;Q45U08_YEAST
346	STH1_YEAST
347	YIN0_YEAST;Q45U13_YEAST
348	RL16A_YEAST;Q45U16_YEAST
349	TM108_YEAST
350	INV2_YEAST;INV1_YEAST;INV3_YEAST;INV4_YEAST;INV5_YEAST;Q65C74_YEAST
351	YIQ6_YEAST
352	YIS3_YEAST
353	PAN1_YEAST
354	DAL81_YEAST
355	PVH1_YEAST
356	GPX3_YEAST
357	GST1_YEAST
358	OST1_YEAST
359	YJB0_YEAST
360	VTC4_YEAST
361	TCPG_YEAST
362	BBC1_YEAST
363	GRP78_YEAST
364	NSP1_YEAST
365	TIM54_YEAST
366	BNA3_YEAST
367	SC160_YEAST
368	EXO70_YEAST;Q66R70_YEAST
369	TRNL_YEAST
370	TCPH_YEAST
371	YJM3_YEAST
372	PYR1_YEAST
373	DS1P1_YEAST
374	IF4A_YEAST

375 CIS3_YEAST
376 HAL5_YEAST
377 YJR1_YEAST
378 RL17B_YEAST;RL17A_YEAST
379 MNN5_YEAST
380 ELO1_YEAST
381 IF2A_YEAST;Q05836_YEAST;Q6Q5P0_YEAST
382 G3P2_YEAST
383 ILV3_YEAST
384 3HAO_YEAST
385 RAV1_YEAST
386 DPOD3_YEAST
387 OSM1_YEAST
388 PTK2_YEAST;Q66R63_YEAST
389 ARP3_YEAST
390 TOR1_YEAST
391 PEM2_YEAST
392 SODC_YEAST
393 ADK_YEAST
394 YJ81_YEAST
395 ATPB_YEAST
396 RS5_YEAST
397 MNS1_YEAST
398 PMT4_YEAST
399 BCA2_YEAST
400 LAC1_YEAST
401 UFD4_YEAST
402 MAOM_YEAST
403 UGPA1_YEAST
404 PTM1_YEAST
405 YKF4_YEAST
406 NU120_YEAST
407 ALF_YEAST
408 YKG3_YEAST
409 NDK_YEAST
410 LHS1_YEAST
411 VATC_YEAST
412 EF1G2_YEAST
413 RRP14_YEAST
414 MDHM_YEAST;Q6Q5N4_YEAST
415 YJU3_YEAST
416 YKK0_YEAST
417 AMPL_YEAST
418 BAF1_YEAST
419 RAD27_YEAST
420 APN1_YEAST
421 SBA1_YEAST
422 PGM1_YEAST
423 AVT3_YEAST
424 PMG1_YEAST
425 PIR1_YEAST
426 KKQ8_YEAST
427 EBP2_YEAST
428 SN114_YEAST
429 FAS1_YEAST;Q05747_YEAST

430 MIA40_YEAST
431 YKT6_YEAST
432 XPOT_YEAST
433 SAC1_YEAST
434 VPS1_YEAST
435 YKZ6_YEAST
436 YKR18_YEAST
437 SPO14_YEAST
438 PET10_YEAST
439 GLG1_YEAST
440 GPT2_YEAST
441 YK54_YEAST;Q6Q573_YEAST
442 NU133_YEAST
443 SRP40_YEAST
444 DNM1_YEAST
445 ORC3_YEAST
446 BPT1_YEAST
447 YL023_YEAST
448 HSP72_YEAST
449 HS104_YEAST
450 GPI13_YEAST
451 PRP19_YEAST;Q8NJV2_YEAST
452 VPS13_YEAST
453 YBT1_YEAST
454 JLP1_YEAST
455 AATC_YEAST
456 RL15A_YEAST;RL15B_YEAST
457 RIC1_YEAST
458 FRE8_YEAST
459 GLYC_YEAST
460 SYFB_YEAST;Q6B2F2_YEAST
461 RL22A_YEAST
462 KIN2_YEAST
463 Q05382_YEAST;MDN1_YEAST
464 YL108_YEAST
465 AHP1_YEAST
466 CFT2_YEAST;Q7LI77_YEAST
467 YPS1_YEAST
468 KICH_YEAST
469 PEP3_YEAST
470 STM1_YEAST
471 ACS2_YEAST
472 UBIQ_YEAST;RS37_YEAST;Q07188_YEAST;RL40_YEAST
473 CBF5_YEAST
474 NOP56_YEAST
475 HRD3_YEAST
476 PPID_YEAST
477 CCC1_YEAST
478 YL225_YEAST
479 AMPM1_YEAST
480 RCK2_YEAST
481 YL253_YEAST
482 YL257_YEAST
483 GYS2_YEAST
484 YPT6_YEAST

485	DBP9_YEAST
486	CHIT_YEAST
487	YL287_YEAST
488	SEC72_YEAST
489	GSP1_YEAST
490	YL301_YEAST
491	MET17_YEAST
492	ACON_YEAST
493	PEX30_YEAST
494	RL38_YEAST
495	TMA10_YEAST
496	CHS5_YEAST
497	NUP2_YEAST
498	FKS1_YEAST
499	TAL1_YEAST
500	ILV5_YEAST;Q02340_YEAST;Q02341_YEAST
501	PUR8_YEAST
502	VAC14_YEAST
503	UTP21_YEAST
504	VIP1_YEAST
505	YL419_YEAST
506	PYRC_YEAST
507	CORO_YEAST
508	SEN1_YEAST;Q7LIE9_YEAST
509	PP2B1_YEAST
510	OAT_YEAST
511	SEC39_YEAST
512	RS3A_YEAST
513	HMDH2_YEAST;Q6B2D0_YEAST
514	PSP2_YEAST
515	APT1_YEAST
516	TSA1_YEAST;Q02552_YEAST
517	PRP39_YEAST;Q6B218_YEAST
518	GSF2_YEAST
519	RS3B_YEAST
520	POB3_YEAST
521	DAK1_YEAST
522	TCB3_YEAST
523	RL6A_YEAST
524	HMDH1_YEAST
525	TBA1_YEAST;TBA3_YEAST
526	TSL1_YEAST
527	NDI1_YEAST
528	PHO84_YEAST
529	HMCS_YEAST
530	YMN1_YEAST;Q6Q5K5_YEAST
531	TIF31_YEAST
532	SUB1_YEAST
533	ERB1_YEAST
534	ARGJ_YEAST
535	PDS5_YEAST
536	ADH3_YEAST
537	YMX6_YEAST;Q6B304_YEAST
538	YPK2_YEAST
539	PGM2_YEAST

540 ILVB_YEAST
541 HFD1_YEAST
542 GBLP_YEAST
543 PUR92_YEAST
544 NCBP1_YEAST
545 DHR1_YEAST
546 RL13B_YEAST
547 NDH1_YEAST
548 YM27_YEAST
549 ALDH3_YEAST;ALDH2_YEAST
550 DDR48_YEAST
551 GCSP_YEAST
552 RL36A_YEAST
553 TOM40_YEAST
554 KIME_YEAST
555 SCJ1_YEAST
556 RNA1_YEAST
557 BCH1_YEAST
558 YHM2_YEAST
559 RL20_YEAST
560 YM81_YEAST
561 DCE_YEAST
562 IF1A_YEAST
563 RSN1_YEAST
564 PYRX_YEAST;Q05774_YEAST
565 NGL2_YEAST
566 HAS1_YEAST
567 GATH_YEAST
568 LCB1_YEAST
569 ATM1_YEAST
570 SCW10_YEAST
571 GAS1_YEAST
572 EIF3C_YEAST
573 YNB0_YEAST
574 PUB1_YEAST
575 HDA1_YEAST
576 SAM50_YEAST;Q6IEH7_YEAST
577 IDH1_YEAST
578 PRA1_YEAST
579 VDAC1_YEAST
580 ARP5_YEAST
581 NOP2_YEAST;Q6B1M0_YEAST
582 SUN4_YEAST
583 RL9B_YEAST
584 ODP2_YEAST
585
TOP2_YEAST;Q07114_YEAST;Q8TF86_YEAST;Q8TG43_YEAST;Q8TG44_YEAST;Q8TG46_YEAST;Q8TG47_YEAST;Q8TG53_YEAST;Q8TG56_YEAST;Q8TG58_YEAST
586 RS7B_YEAST;Q45TZ8_YEAST
587 RAS2_YEAST
588 LEU1_YEAST
589 TOM70_YEAST
590 CPT1_YEAST
591 YNN2_YEAST
592 FKBP_YEAST

593 NAM9_YEAST
594 THO2_YEAST
595 CBK1_YEAST
596 MDG1_YEAST
597 WHI3_YEAST
598 PDR16_YEAST
599 MED16_YEAST
600 BLH1_YEAST
601 SLA2_YEAST
602 SYC_YEAST
603 SIP3_YEAST
604 DSL1_YEAST
605 PIK1_YEAST
606 GYP3_YEAST
607 KRI1_YEAST
608 YN53_YEAST
609 LEM3_YEAST
610 YN86_YEAST
611 DUS2_YEAST
612 ACAC_YEAST
613 ARE2_YEAST
614 YN034_YEAST
615 SIN3_YEAST
616 RCL1_YEAST
617 KCC2_YEAST;Q05436_YEAST
618 GAS5_YEAST
619 RLA2_YEAST
620 A6N9K9_YEAST;GSHB_YEAST
621 BRX1_YEAST
622 MSH2_YEAST
623 SYWC_YEAST;Q6B1Y2_YEAST
624 ZEO1_YEAST
625 IF4E_YEAST
626 RIB4_YEAST
627 CTR9_YEAST
628 GRE2_YEAST
629 ZPS1_YEAST
630 YSP3_YEAST
631 SGT2_YEAST
632 2A5D_YEAST
633 CH10_YEAST;Q6B158_YEAST
634 DBP5_YEAST
635 XRN2_YEAST
636 RL3_YEAST
637 UFE1_YEAST
638 OST3_YEAST
639 TCB1_YEAST
640 VPS21_YEAST
641 RPIA_YEAST
642 OST2_YEAST
643 YO112_YEAST
644 PUR6_YEAST
645 IDH2_YEAST
646 SMP3_YEAST
647 RPB2_YEAST;Q6E5W5_YEAST;Q6JEI2_YEAST

648 PDR5_YEAST
649 DDP1_YEAST
650 SYQ_YEAST
651 HEMH_YEAST
652 SERC_YEAST
653 BFR1_YEAST
654 RPC2_YEAST
655 MGM1_YEAST
656 STE13_YEAST
657 ODC2_YEAST
658 YO227_YEAST
659 WTM1_YEAST
660 AB140_YEAST
661 DGA1_YEAST
662 SEC63_YEAST
663 HRK1_YEAST
664 SNF2_YEAST
665 MBF1_YEAST
666 ISW2_YEAST
667 NOP58_YEAST
668 SNC2_YEAST
669 VATE_YEAST
670 KRE5_YEAST
671 RPA1_YEAST
672 ETFD_YEAST
673 EIF3B_YEAST
674 PSA3_YEAST
675 RS12_YEAST
676 ALDH4_YEAST
677 DHE4_YEAST;DHE5_YEAST
678 ATF1_YEAST;Q6XBT2_YEAST
679 YP009_YEAST
680 RRP12_YEAST
681 IRC15_YEAST
682 VTC3_YEAST
683 MTHR1_YEAST
684 TRM44_YEAST
685 SVL3_YEAST
686 NACB1_YEAST
687 MNN9_YEAST;SDA1_YEAST
688 YP067_YEAST
689 MUK1_YEAST
690 SEC16_YEAST
691 NOG1_YEAST
692 SEC62_YEAST
693 GDE1_YEAST
694 RNY1_YEAST
695 H1_YEAST
696 TBF1_YEAST
697 TAF14_YEAST
698 CARP_YEAST
699 BEM4_YEAST
700 YP183_YEAST
701 UIP4_YEAST
702 HRR25_YEAST

703 SRP72_YEAST
704 SAR1_YEAST
705 YP225_YEAST
706 FAS2_YEAST
707 RUVB2_YEAST
708 IF2B_YEAST
709 RL36B_YEAST
710 MDL2_YEAST
711 SAM3_YEAST
712 RPA2_YEAST
713 AGC1_YEAST
714 YOP1_YEAST
715 IF5_YEAST
716 YP045_YEAST
717 TF2B_YEAST;Q6B148_YEAST
718 PSB5_YEAST
719 COG4_YEAST
720 PIS_YEAST
721 SCD6_YEAST
722 IWS1_YEAST
723 ASNS1_YEAST
724 NCA2_YEAST
725 RHO1_YEAST
726 SEC23_YEAST
727 GDE_YEAST
728 SKI3_YEAST
729 QCR2_YEAST
