# Supplementary material for: Elevated Proteasome Capacity Extends Replicative Lifespan in Saccharomyces cerevisiae
Source: PLoS Genet. 2011 Sep 8;7(9):e1002253. doi: 10.1371/journal.pgen.1002253 (PMC3169524; doi:10.1371/journal.pgen.1002253)
Supplement: Table S4 — Proteins downregulated in rpn4Δ cells with a log2(ratio) <−0.5 relative to WT abundance chloride treatment. (PDF) [file pgen.1002253.s007.pdf]

Table S4: Proteins with log2 >-0.5 fold decreased abundance in *rpn4Δ* cells relative to WT.

1	COX1_YEAST;Q36738_YEAST;Q36739_YEAST;Q36740_YEAST;Q95947_YEAST
2	RMAR_YEAST
3	PMT2_YEAST
4	MYO4_YEAST
5	GIP4_YEAST
6	IF2P_YEAST
7	KPYK1_YEAST
8	AIM2_YEAST
9	ACS1_YEAST;Q66RJ0_YEAST
10	BDH1_YEAST;Q6B208_YEAST
11	ERP1_YEAST;Q6Q5U1_YEAST
12	CACM_YEAST
13	H2A2_YEAST;H2A1_YEAST
14	UTP20_YEAST
15	LONM_YEAST
16	NCL1_YEAST
17	YB029_YEAST
18	AP2A_YEAST
19	PRX1_YEAST
20	NU170_YEAST
21	RL23_YEAST
22	AMPM2_YEAST
23	CND2_YEAST
24	ECM21_YEAST
25	YBK4_YEAST
26	KPC1_YEAST
27	O42833_YEAST;UGA2_YEAST
28	TTP1_YEAST
29	IMB2_YEAST
30	CDS1_YEAST
31	FAT1_YEAST
32	YBQ3_YEAST
33	YBQ6_YEAST
34	HSP26_YEAST
35	ECM33_YEAST
36	C1TM_YEAST
37	VPS15_YEAST
38	PHO88_YEAST
39	CYC8_YEAST
40	SYG_YEAST
41	YBY9_YEAST
42	MAK5_YEAST
43	APD1_YEAST
44	MKAR_YEAST
45	TYR1_YEAST
46	HSP79_YEAST
47	RS6_YEAST
48	G6PI_YEAST
49	KTR4_YEAST
50	KTR3_YEAST;Q6B169_YEAST
51	YBP1_YEAST
52	PYC2_YEAST
53	FAT2_YEAST

54	GLU2A_YEAST
55	OM14_YEAST
56	AROG_YEAST
57	RGD1_YEAST
58	GLYM_YEAST
59	SNF5_YEAST
60	HIS2_YEAST
61	YCE5_YEAST
62	CISY2_YEAST
63	RV161_YEAST
64	ADP1_YEAST
65	RS14B_YEAST;RS14A_YEAST
66	TUP1_YEAST
67	ABP1_YEAST
68	TSC13_YEAST
69	ARP2_YEAST
70	NAT1_YEAST
71	NPC2_YEAST
72	RL31A_YEAST;RL31B_YEAST;Q06739_YEAST
73	RL13A_YEAST
74	RPN6_YEAST
75	TRM3_YEAST
76	NUP84_YEAST
77	KAR_YEAST
78	CDC48_YEAST
79	HOSM_YEAST;Q6B1Y9_YEAST
80	RL35_YEAST
81	COPA_YEAST
82	NOP14_YEAST
83	GLT1_YEAST
84	SEC31_YEAST
85	GLE1_YEAST
86	NHP2_YEAST
87	FMP45_YEAST
88	HSP75_YEAST;Q05833_YEAST;HSP76_YEAST
89	YPD1_YEAST
90	YD237_YEAST
91	TREA_YEAST;Q05216_YEAST
92	YRB1_YEAST
93	RL4B_YEAST
94	GCST_YEAST
95	HEX2_YEAST
96	PST2_YEAST
97	MRH1_YEAST
98	SYKC_YEAST;Q9HE17_YEAST
99	TPIS_YEAST
100	MAK21_YEAST
101	RS13_YEAST
102	PAA1_YEAST
103	SED1_YEAST
104	BMH2_YEAST;BMH1_YEAST
105	TRM1_YEAST;Q9URQ6_YEAST;Q9Y747_YEAST
106	FIMB_YEAST
107	DOP1_YEAST
108	ODO2_YEAST

109 CYPH_YEAST
110 SEC5_YEAST
111 MS116_YEAST
112 GLU2B_YEAST
113 RTN1_YEAST
114 LYS4_YEAST
115 COPB_YEAST
116 MET32_YEAST
117 RSC3_YEAST
118 CYPD_YEAST
119 UBX5_YEAST
120 HXT7_YEAST;HXT6_YEAST
121 GGA1_YEAST
122 ESF1_YEAST
123 YA11B_YEAST;YP12B_YEAST;YN12B_YEAST;YD15B_YEAST;YL14B_YEAST;TY1AB_YEAST;YL12B_YEAST;YE11B_YEAST;Q03970_YEAST;YP11B_YEAST;YP14B_YEAST;YD13B_YEAST;YA11A_YEAST;YD13A_YEAST;TY1A_YEAST;YD15A_YEAST;YO11B_YEAST;YL14A_YEAST;YP14A_YEAST;YN12A_YEAST;YO11A_YEAST
124 ARO10_YEAST
125 YRA1_YEAST
126 EF2_YEAST
127 RV167_YEAST
128 SAC7_YEAST
129 SXM1_YEAST
130 RL12_YEAST
131 NOP3_YEAST
132 GPI17_YEAST
133 RS17B_YEAST;RS17A_YEAST
134 UGO1_YEAST
135 VPS3_YEAST
136 SMT3_YEAST
137 GLRX2_YEAST
138 EUG1_YEAST;Q6B1W0_YEAST
139 YD539_YEAST
140 OSTB_YEAST
141 GLGB_YEAST
142 GEA2_YEAST
143 DPO5_YEAST
144 AFG3_YEAST
145 RPN3_YEAST
146 MED17_YEAST
147 ARB1_YEAST
148 SAHH_YEAST
149 TPA1_YEAST
150 GIP2_YEAST
151 HIS1_YEAST
152 YEP7_YEAST
153 ARG56_YEAST
154 RIR1_YEAST
155 ALDH5_YEAST
156 GET2_YEAST
157 IMB4_YEAST
158 GLO3_YEAST
159 COX15_YEAST

160 TBP_YEAST
161 BEM2_YEAST
162 COG3_YEAST
163 ATC5_YEAST
164 SEC4_YEAST
165 BLM10_YEAST
166 HSP12_YEAST
167 DLDH_YEAST
168 CAF16_YEAST
169 AGX1_YEAST
170 RL22B_YEAST
171 A4URX6_YEAST;A4URX7_YEAST;A4URX8_YEAST;A4URX9_YEAST;A4URY0_YEAST;A4URY1_YEAST;A4URY2_YEAST;A4URY3_YEAST;A4URY4_YEAST;A4URY5_YEAST;ACT_YEAST;Q7Z9V1_YEAST
172 HIS9_YEAST
173 CDC14_YEAST
174 A0SXI4_YEAST;A0SXI5_YEAST;A0SXI6_YEAST;A0SXI7_YEAST;A0SXI9_YEAST;A0SXJ0_YEAST;A0SXJ1_YEAST;MET10_YEAST
175 CPGL_YEAST
176 PDR1_YEAST
177 STT3_YEAST
178 RL30_YEAST
179 MTC2_YEAST
180 YGF9_YEAST
181 YBP2_YEAST
182 YGI2_YEAST;Q6Q536_YEAST
183 G4P1_YEAST
184 RMD9_YEAST
185 SNF4_YEAST
186 NAB2_YEAST
187 YGO0_YEAST
188 HUL5_YEAST
189 MED5_YEAST
190 YIP5_YEAST
191 ATC1_YEAST
192 CLH_YEAST
193 YPT32_YEAST
194 NIF3_YEAST;Q6B164_YEAST
195 YGX8_YEAST
196 SEC15_YEAST
197 SYEC_YEAST
198 DOM3Z_YEAST
199 HXKB_YEAST
200 T2FB_YEAST
201 RS25A_YEAST;RS25B_YEAST
202 UFD1_YEAST
203 EIF2A_YEAST
204 PIL1_YEAST
205 PDC6_YEAST
206 SYV_YEAST
207 SPT6_YEAST
208 UTP8_YEAST
209 NAT2_YEAST
210 PEM1_YEAST

211	IF4F1_YEAST
212	RIR4_YEAST
213	CRH1_YEAST
214	G3P3_YEAST
215	XKS1_YEAST;Q96WW7_YEAST
216	FYV8_YEAST
217	ZPR1_YEAST
218	XPO1_YEAST
219	K6PF1_YEAST
220	SYMC_YEAST
221	BGL2_YEAST
222	RL14B_YEAST;RL14A_YEAST
223	LAG1_YEAST
224	YHB7_YEAST
225	RL8A_YEAST
226	CP51_YEAST
227	SODM_YEAST
228	RL27A_YEAST;RL27B_YEAST
229	SYNC_YEAST
230	RS27B_YEAST;RS27A_YEAST
231	RPN1_YEAST
232	YHJ9_YEAST
233	NCPR_YEAST
234	AAP1_YEAST
235	FSH1_YEAST
236	PANE_YEAST
237	NADE_YEAST
238	NMD2_YEAST
239	YHO0_YEAST
240	HXT5_YEAST
241	SFB3_YEAST
242	TRA1_YEAST
243	GRE3_YEAST
244	CDC12_YEAST
245	YHR2_YEAST
246	LSM12_YEAST
247	EPT1_YEAST
248	ARO9_YEAST
249	SS100_YEAST
250	CDC23_YEAST
251	ELP5_YEAST
252	GPI16_YEAST
253	FDFT_YEAST
254	BCA1_YEAST
255	ACA2_YEAST
256	YID9_YEAST
257	SYG1_YEAST
258	YIF5_YEAST
259	RPN2_YEAST
260	SYTC_YEAST
261	SLM1_YEAST
262	6P21_YEAST
263	ODO1_YEAST;Q45U08_YEAST
264	STH1_YEAST
265	MET18_YEAST

266 YIN0_YEAST;Q45U13_YEAST
267 OM45_YEAST
268 TM108_YEAST
269 YIQ6_YEAST
270 PAN1_YEAST
271 PVH1_YEAST
272 OST1_YEAST
273 BBC1_YEAST
274 NU192_YEAST
275 NSP1_YEAST
276 G3P1_YEAST
277 BNA3_YEAST
278 MPM1_YEAST
279 YJH0_YEAST
280 SC160_YEAST
281 IML2_YEAST
282 OTC_YEAST
283 UTP10_YEAST
284 PYR1_YEAST
285 DS1P1_YEAST
286 IF4A_YEAST
287 INO1_YEAST
288 VPS35_YEAST
289 HAL5_YEAST
290 YJR1_YEAST
291 RL17B_YEAST;RL17A_YEAST
292 MNN5_YEAST
293 IF2A_YEAST;Q05836_YEAST;Q6Q5P0_YEAST
294 3HAO_YEAST
295 URB2_YEAST
296 HSP77_YEAST
297 OSM1_YEAST
298 ARP3_YEAST
299 DOHH_YEAST
300 CARB_YEAST
301 YJ81_YEAST
302 STE24_YEAST
303 ATPB_YEAST
304 RS5_YEAST
305 MNS1_YEAST
306 KAPS_YEAST
307 LAC1_YEAST
308 ATP7_YEAST
309 PTM1_YEAST
310 ALF_YEAST
311 YKG3_YEAST
312 YET1_YEAST
313 YKH1_YEAST
314 LHS1_YEAST
315 VATC_YEAST
316 EF1G2_YEAST
317 RRP14_YEAST
318 YJU3_YEAST
319 YKK0_YEAST
320 GFA1_YEAST

321 BAF1_YEAST
322 APN1_YEAST
323 AP1B1_YEAST
324 PMG1_YEAST
325 KKQ8_YEAST
326 EBP2_YEAST
327 FAS1_YEAST;Q05747_YEAST
328 MIA40_YEAST
329 YKT6_YEAST
330 EAP1_YEAST
331 XPOT_YEAST
332 VPS1_YEAST
333 RSC4_YEAST
334 PRY2_YEAST
335 YPT52_YEAST
336 SPO14_YEAST
337 UTH1_YEAST
338 GPT2_YEAST
339 GTO2_YEAST
340 MTD1_YEAST
341 TGL4_YEAST
342 SRP40_YEAST
343 MLP1_YEAST
344 ORC3_YEAST
345 YL023_YEAST
346 VPS13_YEAST
347 RL8B_YEAST
348 MHT1_YEAST
349 AATC_YEAST
350 RIC1_YEAST
351 PDC1_YEAST
352 GLYC_YEAST
353 RL22A_YEAST
354 MED14_YEAST
355 RL10_YEAST
356 IOC2_YEAST
357 Q05382_YEAST;MDN1_YEAST
358 YL108_YEAST
359 AHP1_YEAST
360 YPS1_YEAST
361 STM1_YEAST
362 ACS2_YEAST
363 UBIQ_YEAST;RS37_YEAST;Q07188_YEAST;RL40_YEAST
364 METK1_YEAST
365 HRD3_YEAST
366 PPID_YEAST
367 YL225_YEAST
368 RCK2_YEAST
369 HSP60_YEAST
370 YPT6_YEAST
371 YL287_YEAST
372 GSP1_YEAST
373 YL301_YEAST
374 MET17_YEAST
375 STT4_YEAST

376 RL38_YEAST
377 TMA10_YEAST
378 SGD1_YEAST
379 RLA0_YEAST
380 FKS1_YEAST
381 ELO3_YEAST
382 STE23_YEAST;Q86ZS8_YEAST
383 CCW14_YEAST
384 VPS33_YEAST
385 SKI2_YEAST
386 YL419_YEAST
387 PYRC_YEAST
388 CORO_YEAST
389 SEN1_YEAST;Q7LIE9_YEAST
390 OAT_YEAST
391 HMDH2_YEAST;Q6B2D0_YEAST
392 YPT7_YEAST
393 PSP2_YEAST
394 APT1_YEAST
395 AMPD_YEAST
396 RS3B_YEAST
397 HMDH1_YEAST
398 TSL1_YEAST
399 NDI1_YEAST
400 HMCS_YEAST
401 MSC1_YEAST
402 YMN1_YEAST;Q6Q5K5_YEAST
403 MVP1_YEAST
404 TIF31_YEAST
405 YMR7_YEAST
406 RNA14_YEAST
407 ARGJ_YEAST
408 ABF2_YEAST
409 ADH3_YEAST
410 YPK2_YEAST
411 KU80_YEAST
412 ILVB_YEAST
413 HFD1_YEAST
414 NCBP1_YEAST
415 OSTD_YEAST
416 YM27_YEAST
417 ALDH3_YEAST;ALDH2_YEAST
418 SSO2_YEAST;SSO1_YEAST
419 GCSP_YEAST
420 YM54_YEAST
421 KIME_YEAST
422 SCJ1_YEAST
423 YM71_YEAST
424 RRP5_YEAST
425 RNA1_YEAST
426 RL20_YEAST
427 LCF4_YEAST
428 YM81_YEAST
429 YM8B_YEAST
430 IF1A_YEAST

431 TPS3_YEAST
432 RSN1_YEAST
433 PYRX_YEAST;Q05774_YEAST
434 HAS1_YEAST
435 GATH_YEAST
436 LCB1_YEAST
437 LIP1_YEAST
438 PUR1_YEAST
439 IMB3_YEAST
440 PUB1_YEAST
441 HDA1_YEAST
442 SAM50_YEAST;Q6IEH7_YEAST
443 VDAC1_YEAST
444 RL9B_YEAST
445 RL16B_YEAST
446 EOS1_YEAST
447 RAS2_YEAST
448 LEU1_YEAST
449 DCP2_YEAST
450 YNN4_YEAST
451 NAM9_YEAST
452 KC12_YEAST
453 YGP1_YEAST
454 CBK1_YEAST
455 BNI5_YEAST
456 RS3_YEAST
457 YNS1_YEAST
458 YNU8_YEAST
459 RAP1_YEAST
460 PURA_YEAST
461 SLA2_YEAST
462 DSL1_YEAST
463 COPG_YEAST
464 GYP3_YEAST
465 RS19B_YEAST;RS19A_YEAST
466 YN53_YEAST
467 LEM3_YEAST
468 CISY1_YEAST
469 YN86_YEAST
470 DUS2_YEAST
471 YN8B_YEAST
472 ALG12_YEAST
473 DBP6_YEAST
474 BRE5_YEAST
475 SIN3_YEAST
476 KCC2_YEAST;Q05436_YEAST
477 TLG2_YEAST;Q6B162_YEAST
478 RLA2_YEAST
479 A6N9K9_YEAST;GSHB_YEAST
480 PSH1_YEAST
481 MDM20_YEAST
482 ZEO1_YEAST
483 RIB4_YEAST
484 GRE2_YEAST
485 SGT2_YEAST

486 CH10_YEAST;Q6B158_YEAST
487 SHE4_YEAST
488 DBP5_YEAST
489 TCB1_YEAST
490 VPS21_YEAST
491 RPIA_YEAST
492 RS7A_YEAST
493 NUP1_YEAST
494 INP53_YEAST
495 PUR6_YEAST
496 ORT1_YEAST
497 RPB2_YEAST;Q6E5W5_YEAST;Q6JEI2_YEAST
498 SYQ_YEAST
499 SERC_YEAST
500 GSP2_YEAST
501 BFR1_YEAST
502 NOC2_YEAST
503 RPC2_YEAST
504 MGM1_YEAST
505 STE13_YEAST
506 ODC2_YEAST
507 WTM1_YEAST
508 RL33B_YEAST
509 DGA1_YEAST
510 SEC63_YEAST
511 HRK1_YEAST
512 MBF1_YEAST
513 VATE_YEAST
514 YO352_YEAST
515 ETFD_YEAST
516 EIF3B_YEAST
517 PSA3_YEAST
518 RS12_YEAST
519 ALDH4_YEAST
520 DHE4_YEAST;DHE5_YEAST
521 IRC15_YEAST
522 TRM44_YEAST
523 PMA2_YEAST
524 NACB1_YEAST
525 PDR12_YEAST
526 ALDH6_YEAST
527 MUK1_YEAST
528 MOT1_YEAST
529 BRO1_YEAST
530 NOG1_YEAST
531 SEC62_YEAST
532 YP105_YEAST
533 GDE1_YEAST
534 RL5_YEAST
535 OYE3_YEAST;Q6B154_YEAST
536 UIP4_YEAST
537 CBP3_YEAST
538 BMS1_YEAST
539 YP225_YEAST
540 NEW1_YEAST;Q8TFA0_YEAST;Q8TFP3_YEAST

541	RUVB2_YEAST
542	IF2B_YEAST
543	HSP82_YEAST
544	SRP68_YEAST
545	YP260_YEAST
546	MDL2_YEAST
547	SAM3_YEAST
548	RPA2_YEAST
549	YME1_YEAST
550	YP045_YEAST
551	TKT1_YEAST
552	SYG2_YEAST
553	PSB5_YEAST
554	SCD6_YEAST
555	NOC4_YEAST
556	PHSG_YEAST
557	SEC23_YEAST
558	QCR2_YEAST
