# Supplementary material for: Elevated Proteasome Capacity Extends Replicative Lifespan in Saccharomyces cerevisiae
Source: PLoS Genet. 2011 Sep 8;7(9):e1002253. doi: 10.1371/journal.pgen.1002253 (PMC3169524; doi:10.1371/journal.pgen.1002253)
Supplement: Table S5 — Proteins upregulated in ubr2Δ cells with a log2(ratio) > 0.5 relative to WT abundance chloride treatment. (PDF) [file pgen.1002253.s008.pdf]

Table S5: Proteins with log2 >0.5 fold increased abundance in *ubr2Δ* cells relative to WT.

1	COX1_YEAST;Q36738_YEAST;Q36739_YEAST;Q36740_YEAST;Q95947_YEAST
2	KAB7_YEAST
3	MAK16_YEAST
4	GIP4_YEAST
5	IF2P_YEAST
6	KPYK1_YEAST
7	ERV46_YEAST
8	PTA1_YEAST
9	CALX_YEAST
10	BDH1_YEAST;Q6B208_YEAST
11	ERP1_YEAST;Q6Q5U1_YEAST
12	PUR7_YEAST
13	IMDH2_YEAST;IMDH3_YEAST;IMDH1_YEAST
14	H2B2_YEAST;H2B1_YEAST
15	H2A2_YEAST;H2A1_YEAST
16	SLA1_YEAST
17	PEP1_YEAST
18	NCL1_YEAST
19	LSM2_YEAST
20	RL19_YEAST
21	ADT2_YEAST
22	URA7_YEAST
23	QCR1_YEAST
24	EDE1_YEAST
25	YBF5_YEAST;Q6B2V4_YEAST
26	PRX1_YEAST
27	RS8_YEAST
28	SYIC_YEAST
29	CDC27_YEAST
30	RL23_YEAST
31	CND2_YEAST
32	O13449_YEAST;ATPA_YEAST
33	O42833_YEAST;UGA2_YEAST
34	O13590_YEAST;H3_YEAST;Q12681_YEAST
35	IPYR_YEAST
36	ATPG_YEAST
37	QOR_YEAST
38	YBQ3_YEAST
39	PRP6_YEAST
40	YBQ6_YEAST
41	HSP26_YEAST
42	ECM33_YEAST
43	C1TM_YEAST
44	ADT3_YEAST
45	IST2_YEAST
46	PPA3_YEAST
47	VPS15_YEAST
48	CYC8_YEAST
49	SYG_YEAST
50	IRA1_YEAST
51	MAK5_YEAST
52	ERF1_YEAST
53	APD1_YEAST

54	HSP79_YEAST
55	RL21A_YEAST;RL21B_YEAST
56	G6PI_YEAST
57	YBP1_YEAST
58	PYC2_YEAST
59	GLU2A_YEAST
60	OM14_YEAST
61	RGD1_YEAST
62	GLYM_YEAST
63	HIS2_YEAST
64	SRO9_YEAST
65	PDI_YEAST
66	PBN1_YEAST
67	SPB1_YEAST
68	YCF7_YEAST
69	YCP4_YEAST
70	CISY2_YEAST
71	RV161_YEAST
72	PGK_YEAST
73	CWH43_YEAST
74	SYP1_YEAST
75	RS14B_YEAST;RS14A_YEAST
76	THRC_YEAST;Q66RI3_YEAST
77	CTR86_YEAST
78	ATG15_YEAST
79	PAT1_YEAST
80	TUP1_YEAST
81	OSH2_YEAST
82	ARP2_YEAST
83	NPC2_YEAST
84	PLSC_YEAST;Q6B238_YEAST
85	PBP4_YEAST
86	MPG1_YEAST
87	TRM3_YEAST
88	HNT1_YEAST
89	CDC48_YEAST
90	HOSM_YEAST;Q6B1Y9_YEAST
91	GDIR_YEAST
92	RL35_YEAST
93	ARF2_YEAST;ARF1_YEAST
94	DHH1_YEAST
95	FADH_YEAST
96	DLD1_YEAST
97	HOSC_YEAST
98	RTN2_YEAST
99	GLE1_YEAST
100	NHP2_YEAST
101	DHE2_YEAST
102	HSP75_YEAST;Q05833_YEAST;HSP76_YEAST
103	YD237_YEAST
104	AAD14_YEAST;AAD4_YEAST;AAD10_YEAST;AAD6_YEAST;Q6B1B9_YEAST
105	YRB1_YEAST
106	MAF1_YEAST
107	SNQ2_YEAST
108	GCST_YEAST

109 SYSC_YEAST
110 VPS54_YEAST
111 HEX2_YEAST
112 AROF_YEAST
113 PST1_YEAST
114 OS9_YEAST
115 MAK21_YEAST
116 LCB2_YEAST
117 PAA1_YEAST
118 SC61G_YEAST
119 RLI1_YEAST
120 YH11B_YEAST;YP13B_YEAST;YD14B_YEAST;YD11B_YEAST;YD12B_YEAST;YB12B_YEAST;YE12B_YEAST;YL11B_YEAST;YG13B_YEAST;YM14B_YEAST;YO12B_YEAST;YM11B_YEAST;YH11A_YEAST;YB12A_YEAST;YD11A_YEAST;YD12A_YEAST;YD14A_YEAST;YO12A_YEAST;YM11A_YEAST
121 BMH2_YEAST;BMH1_YEAST
122 ARX1_YEAST
123 TRS85_YEAST
124 KIN1_YEAST
125 HPR1_YEAST
126 DOP1_YEAST
127 ODO2_YEAST
128 CYPH_YEAST
129 SEC5_YEAST
130 STB3_YEAST
131 HSP42_YEAST
132 ERF3_YEAST;Q6Q7I1_YEAST;Q6Q7I2_YEAST;Q6Q7I3_YEAST;Q6Q7I4_YEAST;Q6Q7I6_YEAST;Q8TFA9_YEAST;Q8TFB8_YEAST;Q8TFQ8_YEAST;Q8TFQ9_YEAST;Q8TFR0_YEAST;Q8TFR1_YEAST;Q8TFR3_YEAST;Q8TFR4_YEAST;Q8TFR6_YEAST;Q8TFR7_YEAST;Q8TFR8_YEAST;Q8TFR9_YEAST;Q96TJ4_YEAST;Q96TL8_YEAST;Q96TM0_YEAST;Q96TQ9_YEAST;Q96UI8_YEAST;Q96UI9_YEAST;Q96UJ0_YEAST;Q96UJ1_YEAST;Q96UJ2_YEAST;Q96UJ4_YEAST;Q96UJ5_YEAST;Q96UJ6_YEAST;Q96UJ7_YEAST;Q96UJ8_YEAST;Q9HGV1_YEAST
133 SCC2_YEAST
134 TCPZ_YEAST
135 AHA1_YEAST
136 GLU2B_YEAST
137 RTN1_YEAST
138 COPB_YEAST
139 MNN10_YEAST
140 MET32_YEAST
141 AKR1_YEAST
142 SUR2_YEAST
143 RSC3_YEAST
144 CYPD_YEAST
145 SSF1_YEAST;SSF2_YEAST
146 SKP1_YEAST
147 UBX5_YEAST
148 HXT7_YEAST;HXT6_YEAST
149 HXT3_YEAST
150 PAL1_YEAST
151 EF2_YEAST
152 HPRT_YEAST
153 RL12_YEAST
154 RPN9_YEAST

155 NOP3_YEAST
156 PPZ2_YEAST
157 RS17B_YEAST;RS17A_YEAST
158 UTP6_YEAST
159 TOM1_YEAST
160 HEH2_YEAST
161 UGO1_YEAST
162 SNF1_YEAST
163 KRE2_YEAST
164 METK2_YEAST;Q6B194_YEAST
165 SMT3_YEAST
166 EUG1_YEAST;Q6B1W0_YEAST
167 YD539_YEAST
168 OSTB_YEAST
169 GLGB_YEAST
170 GEA2_YEAST
171 UCRI_YEAST
172 ATC6_YEAST
173 IF5A2_YEAST
174 DPO5_YEAST
175 AGM1_YEAST
176 PRTB_YEAST
177 GPA2_YEAST
178 YEL4_YEAST
179 ARB1_YEAST
180 CAJ1_YEAST
181 AK_YEAST
182 HIS1_YEAST
183 THO1_YEAST
184 ARG56_YEAST
185 RS24_YEAST
186 YEQ7_YEAST
187 A0SXJ4_YEAST;SER33_YEAST;SERA_YEAST
188 THDH_YEAST
189 METE_YEAST;Q27JJ6_YEAST;Q27JK0_YEAST
190 HSP74_YEAST;HSP73_YEAST
191 NU157_YEAST
192 IMB4_YEAST
193 SCS2_YEAST
194 KC13_YEAST
195 RSP5_YEAST
196 COX15_YEAST
197 UBP3_YEAST
198 BEM2_YEAST
199 ODPA_YEAST
200 SEC4_YEAST
201 HSP12_YEAST
202 DLDH_YEAST
203 RL22B_YEAST
204 YPT1_YEAST
205 PMM_YEAST
206 EMP47_YEAST
207 SNZ3_YEAST;SNZ2_YEAST;SNZ1_YEAST
208 NIC96_YEAST
209 YFI6_YEAST

210 CDC14_YEAST
211 A0SXI4_YEAST;A0SXI5_YEAST;A0SXI6_YEAST;A0SXI7_YEAST;A0SXI9_YEAST;A0SXJ0_YEAST;A0SXJ1_YEAST;MET10_YEAST
212 RL2_YEAST
213 CPGL_YEAST
214 ATC2_YEAST
215 PMA1_YEAST
216 LEUC_YEAST
217 ERG4_YEAST;Q6B175_YEAST
218 PDR1_YEAST
219 PDR6_YEAST
220 STT3_YEAST
221 RL24A_YEAST
222 PNC1_YEAST
223 ERV14_YEAST
224 YGF9_YEAST
225 PYC1_YEAST
226 SGF73_YEAST
227 YGI2_YEAST;Q6Q536_YEAST
228 RL28_YEAST
229 G4P1_YEAST
230 PRP43_YEAST
231 NAB2_YEAST
232 ITC1_YEAST
233 HUL5_YEAST
234 RL9A_YEAST
235 AROC_YEAST
236 MED5_YEAST
237 YIP5_YEAST
238 XRN1_YEAST
239 STR3_YEAST
240 GCN1_YEAST
241 KEX1_YEAST
242 SPT16_YEAST
243 PUR2_YEAST;Q6B1U4_YEAST
244 SYEC_YEAST
245 RL26B_YEAST;RL26A_YEAST
246 ORM1_YEAST;Q45U45_YEAST
247 TAL2_YEAST;Q45U40_YEAST;Q6Q5P8_YEAST
248 UFD1_YEAST
249 PUR4_YEAST;Q45U25_YEAST
250 EI2BD_YEAST
251 PIL1_YEAST
252 CATT_YEAST
253 GYP2_YEAST
254 SPT6_YEAST
255 ASNS2_YEAST;Q6B272_YEAST
256 YG35_YEAST
257 YG36_YEAST
258 UTP8_YEAST
259 YG3A_YEAST
260 NSR1_YEAST
261 IF4F1_YEAST
262 CLC1_YEAST

263	RIR4_YEAST
264	CRH1_YEAST
265	XKS1_YEAST;Q96WW7_YEAST
266	FYV8_YEAST
267	XPO1_YEAST
268	HSV2_YEAST
269	AP3B_YEAST
270	SYMC_YEAST
271	SLH1_YEAST
272	BGL2_YEAST
273	ZUO1_YEAST
274	RL14B_YEAST;RL14A_YEAST
275	LAG1_YEAST
276	RS20_YEAST
277	RL8A_YEAST
278	SSBP1_YEAST
279	GPA1_YEAST
280	CP51_YEAST
281	SODM_YEAST
282	RL27A_YEAST;RL27B_YEAST
283	YHH7_YEAST
284	RS27B_YEAST;RS27A_YEAST
285	KHSE_YEAST
286	RPN1_YEAST
287	NCPR_YEAST
288	AAP1_YEAST
289	FSH1_YEAST
290	YHL4_YEAST;RSC30_YEAST
291	PANE_YEAST
292	SSZ1_YEAST
293	HXT5_YEAST
294	GRE3_YEAST
295	GGA2_YEAST
296	YHR2_YEAST
297	LSM12_YEAST
298	EPT1_YEAST
299	ECM14_YEAST
300	RL44_YEAST
301	YHU6_YEAST
302	KEL1_YEAST;Q6B2C7_YEAST
303	CDC23_YEAST
304	KOG1_YEAST
305	ELP5_YEAST
306	GPI16_YEAST
307	NACA_YEAST
308	YH02_YEAST
309	BCA1_YEAST
310	YH09_YEAST;Q6Q5R3_YEAST
311	VID28_YEAST
312	SSM4_YEAST
313	YID9_YEAST
314	NCB5R_YEAST
315	MET30_YEAST
316	MMF1_YEAST
317	RL34B_YEAST;RL34A_YEAST

318 GPP1_YEAST
319 YIF5_YEAST
320 RPN2_YEAST
321 YII3_YEAST
322 SLM1_YEAST
323 MOB1_YEAST
324 HIS8_YEAST
325 ODO1_YEAST;Q45U08_YEAST
326 STH1_YEAST
327 GPDM_YEAST;Q6B263_YEAST
328 YIQ6_YEAST
329 PAN1_YEAST
330 DAL81_YEAST
331 PVH1_YEAST
332 LYS1_YEAST
333 GST1_YEAST
334 VTC4_YEAST
335 TCPG_YEAST
336 BBC1_YEAST
337 RIR2_YEAST
338 NU192_YEAST
339 MTR4_YEAST
340 BNA3_YEAST
341 NET1_YEAST
342 SC160_YEAST
343 IML2_YEAST
344 TRNL_YEAST
345 YJM3_YEAST
346 YAK1_YEAST
347 INO1_YEAST
348 HAL5_YEAST
349 FPPS_YEAST
350 RL17B_YEAST;RL17A_YEAST
351 MNN11_YEAST;Q6B221_YEAST
352 LAA1_YEAST
353 AP2B_YEAST;Q05160_YEAST;Q05162_YEAST
354 DENR_YEAST
355 ILV3_YEAST
356 RAV1_YEAST
357 NUP85_YEAST
358 DPOD3_YEAST
359 HSP77_YEAST
360 YJ16_YEAST
361 OSM1_YEAST
362 PTK2_YEAST;Q66R63_YEAST
363 TOR1_YEAST
364 DOHH_YEAST
365 HOC1_YEAST;Q6B2A3_YEAST
366 MPCP_YEAST
367 JSN1_YEAST
368 SODC_YEAST
369 ADK_YEAST
370 CARB_YEAST
371 YJ81_YEAST
372 MNS1_YEAST

373 PMT4_YEAST
374 LAC1_YEAST
375 UFD4_YEAST
376 URB1_YEAST
377 ATP7_YEAST
378 YET1_YEAST
379 LHS1_YEAST
380 DHR2_YEAST
381 VATC_YEAST
382 RRP14_YEAST
383 MDHM_YEAST;Q6Q5N4_YEAST
384 YJU3_YEAST
385 BAF1_YEAST
386 RAD27_YEAST
387 SBA1_YEAST
388 YPK1_YEAST
389 SDH3_YEAST
390 LTV1_YEAST
391 AVT3_YEAST
392 DHSA_YEAST
393 PIR1_YEAST
394 KKQ8_YEAST
395 EBP2_YEAST
396 FAS1_YEAST;Q05747_YEAST
397 MIA40_YEAST
398 YKT6_YEAST
399 XPOT_YEAST
400 LRC3_YEAST
401 UBA1_YEAST
402 SAC1_YEAST
403 PAP_YEAST
404 FOX2_YEAST
405 PRY2_YEAST
406 YPT52_YEAST
407 YKZ6_YEAST
408 SPO14_YEAST
409 GLG1_YEAST
410 GPT2_YEAST
411 YK54_YEAST;Q6Q573_YEAST
412 GTO2_YEAST
413 MTD1_YEAST
414 RPF2_YEAST
415 SRP40_YEAST
416 BPT1_YEAST
417 SYDC_YEAST
418 SPA2_YEAST
419 HSP72_YEAST
420 GPI13_YEAST
421 YL032_YEAST
422 VPS13_YEAST
423 FPS1_YEAST
424 RL8B_YEAST
425 YBT1_YEAST
426 JLP1_YEAST
427 MHT1_YEAST

428 AATC_YEAST
429 RIC1_YEAST
430 PDC1_YEAST
431 FRE8_YEAST
432 SYFB_YEAST;Q6B2F2_YEAST
433 MED14_YEAST
434 RL10_YEAST
435 GAA1_YEAST
436 IOC2_YEAST
437 KIN2_YEAST
438 Q05382_YEAST;MDN1_YEAST
439 YPS1_YEAST
440 UTP12_YEAST
441 PDC5_YEAST
442 METK1_YEAST
443 MSS51_YEAST
444 HRD3_YEAST
445 SEC13_YEAST
446 PPID_YEAST
447 CCC1_YEAST
448 IFH1_YEAST
449 YL225_YEAST
450 RCK2_YEAST
451 EF3A_YEAST
452 YL253_YEAST
453 YL257_YEAST
454 YPT6_YEAST
455 YL287_YEAST
456 SEC72_YEAST
457 MET17_YEAST
458 ACON_YEAST
459 STT4_YEAST
460 IMH1_YEAST
461 CDC25_YEAST
462 PEX30_YEAST
463 RL38_YEAST
464 TMA10_YEAST
465 RS25B_YEAST;RS25A_YEAST
466 NUP2_YEAST
467 SGD1_YEAST
468 RLA0_YEAST
469 YL352_YEAST
470 ILV5_YEAST;Q02340_YEAST;Q02341_YEAST
471 ELO3_YEAST
472 STE23_YEAST;Q86ZS8_YEAST
473 VPS33_YEAST
474 UTP21_YEAST
475 YL419_YEAST
476 SEN1_YEAST;Q7LIE9_YEAST
477 PP2B1_YEAST
478 OAT_YEAST
479 RS3A_YEAST
480 FKBP4_YEAST
481 HMDH2_YEAST;Q6B2D0_YEAST
482 YPT7_YEAST

483 LGUL_YEAST
484 ERG6_YEAST
485 PSP2_YEAST
486 APT1_YEAST
487 SRC1_YEAST
488 PRP39_YEAST;Q6B218_YEAST
489 GSF2_YEAST
490 TCB3_YEAST
491 RL6A_YEAST
492 HMDH1_YEAST
493 ALO_YEAST
494 NDI1_YEAST
495 PHO84_YEAST
496 RSC9_YEAST
497 MSC1_YEAST
498 ERO1_YEAST;Q6B1N0_YEAST
499 YMN1_YEAST;Q6Q5K5_YEAST
500 MVP1_YEAST
501 YMR7_YEAST
502 SUB1_YEAST
503 NU116_YEAST
504 RNA14_YEAST
505 TVP18_YEAST
506 ABF2_YEAST
507 PDS5_YEAST
508 SEC14_YEAST
509 ADH3_YEAST
510 YMX6_YEAST;Q6B304_YEAST
511 UTP15_YEAST
512 MYO5_YEAST
513 GBLP_YEAST
514 DHR1_YEAST
515 PO152_YEAST
516 NDH1_YEAST
517 YM27_YEAST
518 ALDH3_YEAST;ALDH2_YEAST
519 DDR48_YEAST
520 SPT21_YEAST
521 GCSP_YEAST
522 RL36A_YEAST
523 TOM40_YEAST
524 SCJ1_YEAST
525 YM71_YEAST
526 RRP5_YEAST
527 RS10B_YEAST;RS10A_YEAST
528 BCH1_YEAST
529 RL20_YEAST
530 LCF4_YEAST
531 DCE_YEAST
532 IF1A_YEAST
533 RSN1_YEAST
534 LCB1_YEAST
535 LIP1_YEAST
536 SIS1_YEAST
537 YNB0_YEAST

538 PUB1_YEAST
539 HDA1_YEAST
540 IDH1_YEAST
541 COG6_YEAST
542 LKHA4_YEAST
543 ARP5_YEAST
544 MAS5_YEAST
545 RL9B_YEAST
546 TPM1_YEAST
547 EOS1_YEAST
548 TCB2_YEAST
549 TOP2_YEAST;Q07114_YEAST;Q8TF86_YEAST;Q8TG43_YEAST;Q8TG44_YEAST;Q8TG46_YEAST;Q8TG47_YEAST;Q8TG53_YEAST;Q8TG56_YEAST;Q8TG58_YEAST
550 RS7B_YEAST;Q45TZ8_YEAST
551 FAR11_YEAST
552 CPT1_YEAST
553 YNN2_YEAST
554 YNN4_YEAST
555 NAM9_YEAST
556 THO2_YEAST
557 LSM7_YEAST
558 BNI5_YEAST
559 MDG1_YEAST
560 CHS1_YEAST
561 WHI3_YEAST
562 IES2_YEAST
563 RAP1_YEAST
564 ALG9_YEAST
565 PURA_YEAST
566 PDR16_YEAST
567 MED16_YEAST
568 RPA49_YEAST
569 SIP3_YEAST
570 PIK1_YEAST
571 COPG_YEAST
572 GYP3_YEAST
573 RL18_YEAST
574 RS19B_YEAST;RS19A_YEAST
575 CISY1_YEAST
576 PDAT_YEAST
577 YN86_YEAST
578 YN8B_YEAST
579 YN034_YEAST
580 ARPC2_YEAST
581 DBP6_YEAST
582 BRE5_YEAST
583 SIN3_YEAST
584 RLA2_YEAST
585 PSH1_YEAST
586 MDM20_YEAST
587 BRX1_YEAST
588 MSH2_YEAST
589 SYWC_YEAST;Q6B1Y2_YEAST
590 ZEO1_YEAST

591 IF4E_YEAST
592 RIB4_YEAST
593 NOP8_YEAST
594 GRE2_YEAST
595 2A5D_YEAST
596 CH10_YEAST;Q6B158_YEAST
597 STI1_YEAST
598 CUE5_YEAST
599 XRN2_YEAST
600 RL3_YEAST
601 GYP1_YEAST
602 UFE1_YEAST
603 TCB1_YEAST
604 RPIA_YEAST
605 RS7A_YEAST
606 NUP1_YEAST
607 INP53_YEAST
608 YO112_YEAST
609 GCY_YEAST
610 LEO1_YEAST
611 PUR6_YEAST
612 IDH2_YEAST
613 DDP1_YEAST
614 YO164_YEAST
615 HEMH_YEAST
616 GSP2_YEAST
617 BFR1_YEAST
618 STE13_YEAST
619 YO227_YEAST
620 GRPE_YEAST
621 RL33B_YEAST
622 AB140_YEAST
623 DGA1_YEAST
624 RPN8_YEAST
625 FSF1_YEAST
626 CAF20_YEAST
627 SNF2_YEAST
628 MBF1_YEAST
629 NOP58_YEAST
630 SNC2_YEAST
631 KRE5_YEAST
632 ETFD_YEAST
633 PDE2_YEAST
634 EIF3B_YEAST
635 RS12_YEAST
636 LSP1_YEAST
637 VTC3_YEAST
638 MTHR1_YEAST
639 NACB1_YEAST
640 EF1G1_YEAST
641 PDR12_YEAST
642 ALDH6_YEAST
643 MUK1_YEAST
644 ATPF_YEAST;Q6B1V4_YEAST
645 SEC16_YEAST

646 GSHR_YEAST
647 YP105_YEAST
648 GDE1_YEAST
649 RNY1_YEAST
650 H1_YEAST
651 TBF1_YEAST
652 TAF14_YEAST
653 RL5_YEAST
654 KES1_YEAST;Q6Q5I1_YEAST
655 CARP_YEAST
656 BEM4_YEAST
657 OYE3_YEAST;Q6B154_YEAST
658 YP183_YEAST
659 UIP4_YEAST
660 RL7B_YEAST
661 HRR25_YEAST
662 CBP3_YEAST
663 FAS2_YEAST
664 IF2B_YEAST
665 RL36B_YEAST
666 YP260_YEAST
667 DIM1_YEAST
668 MDL2_YEAST
669 YP045_YEAST
670 TF2B_YEAST;Q6B148_YEAST
671 YP091_YEAST
672 PSB5_YEAST
673 RPN7_YEAST;Q6B2G1_YEAST
674 PIS_YEAST
675 YLH47_YEAST
676 ASNS1_YEAST
677 NCA2_YEAST
678 PHSG_YEAST
679 RHO1_YEAST
680 SKI3_YEAST
681 RPC3_YEAST
682 QCR2_YEAST
