# Supplementary material for: Elevated Proteasome Capacity Extends Replicative Lifespan in Saccharomyces cerevisiae
Source: PLoS Genet. 2011 Sep 8;7(9):e1002253. doi: 10.1371/journal.pgen.1002253 (PMC3169524; doi:10.1371/journal.pgen.1002253)
Supplement: Table S6 — Proteins downregulated in ubr2Δ cells with a log2(ratio) <−0.5 relative to WT abundance chloride treatment. (PDF) [file pgen.1002253.s009.pdf]

Table S6: Proteins with log2 >-1.5 fold decreased abundance in *ubr2Δ* cells relative to WT.

1	COX1_YEAST;Q36738_YEAST;Q36739_YEAST;Q36740_YEAST;Q95947_YEAST
2	2AAA_YEAST
3	KAB7_YEAST
4	GIP4_YEAST
5	IF2P_YEAST
6	KPYK1_YEAST
7	AIM2_YEAST
8	ACS1_YEAST;Q66RJ0_YEAST
9	BDH1_YEAST;Q6B208_YEAST
10	ERP1_YEAST;Q6Q5U1_YEAST
11	CACM_YEAST
12	H2A2_YEAST;H2A1_YEAST
13	UTP20_YEAST
14	NCL1_YEAST
15	LSM2_YEAST
16	RL19_YEAST
17	STU1_YEAST
18	QCR1_YEAST
19	SYIC_YEAST
20	YBI6_YEAST;Q6B2T6_YEAST
21	AMPM2_YEAST
22	CND2_YEAST
23	O13449_YEAST;ATPA_YEAST
24	YBK4_YEAST
25	O42833_YEAST;UGA2_YEAST
26	H4_YEAST
27	O13590_YEAST;H3_YEAST;Q12681_YEAST
28	OLA1_YEAST
29	CDS1_YEAST
30	QOR_YEAST
31	YBQ3_YEAST
32	HSP26_YEAST
33	ECM33_YEAST
34	EIF3A_YEAST
35	SEC18_YEAST
36	ADT3_YEAST
37	PPA5_YEAST;Q06856_YEAST;Q06876_YEAST
38	PHO88_YEAST
39	EF1A_YEAST
40	SYG_YEAST
41	A1E5M4_YEAST;TPS1_YEAST;Q7LIJ9_YEAST
42	IRA1_YEAST
43	ARA1_YEAST
44	APD1_YEAST
45	TOS1_YEAST
46	TYR1_YEAST
47	HSP79_YEAST
48	MCFS2_YEAST;Q9Y714_YEAST
49	RS6_YEAST
50	RL21A_YEAST;RL21B_YEAST
51	KTR3_YEAST;Q6B169_YEAST
52	SDS24_YEAST
53	YBP1_YEAST

54 FAT2_YEAST
55 GLU2A_YEAST
56 OM14_YEAST
57 AROG_YEAST
58 APE3_YEAST
59 HIS2_YEAST
60 SRO9_YEAST
61 GID7_YEAST
62 HXKG_YEAST
63 PDI_YEAST
64 PBN1_YEAST
65 SPB1_YEAST
66 YCF7_YEAST
67 CISY2_YEAST
68 RV161_YEAST
69 SYP1_YEAST
70 RS14B_YEAST;RS14A_YEAST
71 THRC_YEAST;Q66RI3_YEAST
72 TUP1_YEAST
73 ABP1_YEAST
74 NOT1_YEAST
75 FBRL_YEAST
76 OSH2_YEAST
77 NAT1_YEAST
78 RL31A_YEAST;RL31B_YEAST;Q06739_YEAST
79 VAM6_YEAST
80 RPN6_YEAST
81 NUP84_YEAST
82 HNT1_YEAST
83 GDIR_YEAST
84 ARF2_YEAST;ARF1_YEAST
85 COPA_YEAST
86 FADH_YEAST
87 GLT1_YEAST
88 DLD1_YEAST
89 VATA_YEAST;Q86Z53_YEAST;Q86Z73_YEAST;Q874G9_YEAST;Q874H0_YEAST;Q874H1_YEAST;Q874H2_YEAST
90 SEC31_YEAST
91 YHM1_YEAST
92 NHP2_YEAST
93 YPD1_YEAST
94 YRB1_YEAST
95 RL4B_YEAST
96 VPS54_YEAST
97 PST2_YEAST
98 MRH1_YEAST
99 SYKC_YEAST;Q9HE17_YEAST
100 TPIS_YEAST
101 MAK21_YEAST
102 LCB2_YEAST
103 PAA1_YEAST
104 SED1_YEAST
105 RLI1_YEAST
106 ARX1_YEAST

107 TRS85_YEAST
108 TRM1_YEAST;Q9URQ6_YEAST;Q9Y747_YEAST
109 ARO1_YEAST
110 DOP1_YEAST
111 CYPH_YEAST
112 STB3_YEAST
113
ERF3_YEAST;Q6Q7I1_YEAST;Q6Q7I2_YEAST;Q6Q7I3_YEAST;Q6Q7I4_YEAST;Q6Q7I6_YEAST;Q8TFA9_YEAST;Q8TFB8_YEAST;Q8TFQ8_YEAST;Q8TFQ9_YEAST;Q8TFR0_YEAST;Q8TFR1_YEAST;Q8TFR3_YEAST;Q8TFR4_YEAST;Q8TFR6_YEAST;Q8TFR7_YEAST;Q8TFR8_YEAST;Q8TFR9_YEAST;Q96TJ4_YEAST;Q96TL8_YEAST;Q96TM0_YEAST;Q96TQ9_YEAST;Q96UI8_YEAST;Q96UI9_YEAST;Q96UJ0_YEAST;Q96UJ1_YEAST;Q96UJ2_YEAST;Q96UJ4_YEAST;Q96UJ5_YEAST;Q96UJ6_YEAST;Q96UJ7_YEAST;Q96UJ8_YEAST;Q9HGV1_YEAST
114 TCPZ_YEAST
115 AHA1_YEAST
116 RTN1_YEAST
117 MET32_YEAST
118 RSC3_YEAST
119 CYPD_YEAST
120 SSF1_YEAST;SSF2_YEAST
121 UBX5_YEAST
122 HXT7_YEAST;HXT6_YEAST
123 SVF1_YEAST
124 PAL1_YEAST
125
YA11B_YEAST;YP12B_YEAST;YN12B_YEAST;YD15B_YEAST;YL14B_YEAST;TY1AB_YEAST;YL12B_YEAST;YE11B_YEAST;Q03970_YEAST;YP11B_YEAST;YP14B_YEAST;YD13B_YEAST;YA11A_YEAST;YD13A_YEAST;TY1A_YEAST;YD15A_YEAST;YO11B_YEAST;YL14A_YEAST;YP14A_YEAST;YN12A_YEAST;YO11A_YEAST
126 CHI2_YEAST
127 ARO10_YEAST
128 RV167_YEAST
129 HPRT_YEAST
130 RL12_YEAST
131 RPN9_YEAST
132 GPI17_YEAST
133 PPZ2_YEAST
134 UTP6_YEAST
135 RS18_YEAST
136 TOM1_YEAST
137 SNF1_YEAST
138 PEX29_YEAST
139 KRE2_YEAST
140 RIB3_YEAST
141 VPS3_YEAST
142 LCD1_YEAST
143 METK2_YEAST;Q6B194_YEAST
144 YD539_YEAST
145 OSTB_YEAST
146 GLGB_YEAST
147 GEA2_YEAST
148 ATC6_YEAST
149 CRH2_YEAST
150 AFG3_YEAST
151 GPA2_YEAST

152 IF2G_YEAST
153 ARB1_YEAST
154 ERG28_YEAST
155 CAJ1_YEAST
156 TPA1_YEAST
157 AK_YEAST
158 GIP2_YEAST
159 ARG56_YEAST
160 ALDH5_YEAST
161 YEQ7_YEAST
162 YER0_YEAST
163 GET2_YEAST
164 METE_YEAST;Q27JJ6_YEAST;Q27JK0_YEAST
165 HSP74_YEAST;HSP73_YEAST
166 NU157_YEAST
167 IMB4_YEAST
168 SCS2_YEAST
169 GDI1_YEAST
170 COX15_YEAST
171 BEM2_YEAST
172 COG3_YEAST
173 PABP_YEAST
174 SEC4_YEAST
175 DLDH_YEAST
176 PMM_YEAST
177 YFI6_YEAST
178 HIS9_YEAST
179
A0SXI4_YEAST;A0SXI5_YEAST;A0SXI6_YEAST;A0SXI7_YEAST;A0SXI9_YEAST;A0SXJ0_YEAST;A0SXJ1_YEAST;MET10_YEAST
180 RL2_YEAST
181 HXKA_YEAST
182 ATC2_YEAST
183 LEUC_YEAST
184 PDR1_YEAST
185 PDR6_YEAST
186 MTC2_YEAST
187 PNC1_YEAST
188 HEM2_YEAST
189 IF4F2_YEAST
190 YBP2_YEAST
191 RL28_YEAST
192 RMD9_YEAST
193 RS2_YEAST
194 RL1_YEAST
195 AROC_YEAST
196 MED5_YEAST
197 ATC1_YEAST
198 XRN1_YEAST
199 RS26A_YEAST;RS26B_YEAST
200 ARO8_YEAST
201 KEX1_YEAST
202 YPT32_YEAST
203 NIF3_YEAST;Q6B164_YEAST
204 CSE1_YEAST

205 HXKB_YEAST
206 NMA1_YEAST;NMA2_YEAST
207 ORM1_YEAST;Q45U45_YEAST
208 TAL2_YEAST;Q45U40_YEAST;Q6Q5P8_YEAST
209 RL11B_YEAST;RL11A_YEAST
210 PIL1_YEAST
211 PDC6_YEAST
212 SYV_YEAST
213 GYP2_YEAST
214 SPT6_YEAST
215 YG32_YEAST
216 YG35_YEAST
217 NAT2_YEAST
218 RL24B_YEAST
219 CLC1_YEAST
220 RIR4_YEAST
221 SYYC_YEAST
222 XKS1_YEAST;Q96WW7_YEAST
223 C1TC_YEAST
224 6PGD2_YEAST
225 YG5L_YEAST
226 ZUO1_YEAST
227 LAG1_YEAST
228 RS20_YEAST
229 YHC1_YEAST
230 RL8A_YEAST
231 SSBP1_YEAST
232 CBP2_YEAST
233 SODM_YEAST
234 YHG9_YEAST
235 SYNC_YEAST
236 RS27B_YEAST;RS27A_YEAST
237 DAP2_YEAST;Q66R87_YEAST
238 YHJ9_YEAST
239 NCPR_YEAST
240 YHK5_YEAST
241 FSH1_YEAST
242 SSZ1_YEAST
243 NADE_YEAST
244 HXT5_YEAST
245 SFB3_YEAST
246 TRA1_YEAST
247 CDC12_YEAST
248 LSM12_YEAST
249 EPT1_YEAST
250 ARO9_YEAST
251 SS100_YEAST
252 RL44_YEAST
253 YHU6_YEAST
254 KEL1_YEAST;Q6B2C7_YEAST
255 CDC23_YEAST
256 6PGD1_YEAST
257 KOG1_YEAST
258 ELP5_YEAST
259 FDFT_YEAST

260 NACA_YEAST
261 RIX1_YEAST
262 RS4_YEAST
263 VID28_YEAST
264 SSM4_YEAST
265 CSK21_YEAST
266 ACA2_YEAST
267 YID9_YEAST
268 NCB5R_YEAST
269 SYG1_YEAST
270 MMF1_YEAST
271 RL34B_YEAST;RL34A_YEAST
272 YIF5_YEAST
273 YRB2_YEAST
274 ICE2_YEAST;Q6B2T7_YEAST
275 YIJ1_YEAST
276 SLM1_YEAST
277 HIS8_YEAST
278 SIM1_YEAST
279 ODO1_YEAST;Q45U08_YEAST
280 STH1_YEAST
281 YIN0_YEAST;Q45U13_YEAST
282 INV2_YEAST;INV1_YEAST;INV3_YEAST;INV4_YEAST;INV5_YEAST;Q65C74_YEAST
283 LYS1_YEAST
284 GPX3_YEAST
285 BBC1_YEAST
286 GRP78_YEAST
287 NSP1_YEAST
288 TIM54_YEAST
289 MPM1_YEAST
290 YJH0_YEAST
291 TRNL_YEAST
292 OTC_YEAST
293 UTP10_YEAST
294 YJM3_YEAST
295 PYR1_YEAST
296 DS1P1_YEAST
297 INO1_YEAST
298 YJR1_YEAST
299 ELO1_YEAST
300 IF2A_YEAST;Q05836_YEAST;Q6Q5P0_YEAST
301 ILV3_YEAST
302 YJ11B_YEAST;YL13B_YEAST;YM12B_YEAST;YJ12B_YEAST;YG11B_YEAST;YG12B_YEAST;YJ11A_YEAST;YJ12A_YEAST;YG11A_YEAST;YG12A_YEAST
303 RAV1_YEAST
304 NUP85_YEAST
305 DPOD3_YEAST
306 OSM1_YEAST
307 PTK2_YEAST;Q66R63_YEAST
308 TOR1_YEAST
309 PEM2_YEAST
310 MPCP_YEAST
311 ADK_YEAST
312 CARB_YEAST

313 YJ81_YEAST
314 ATPB_YEAST
315 A0SXK5_YEAST;A0SXK6_YEAST;A0SXK7_YEAST;A0SXK8_YEAST;ECM17_YEAST
316 KAPS_YEAST
317 URB1_YEAST
318 UGPA1_YEAST
319 PTM1_YEAST
320 TCTP_YEAST
321 ALF_YEAST
322 YKH1_YEAST
323 RRP14_YEAST
324 MDHM_YEAST;Q6Q5N4_YEAST
325 YJU3_YEAST
326 YKK0_YEAST
327 RAD27_YEAST
328 SBA1_YEAST
329 YPK1_YEAST
330 AP1B1_YEAST
331 SDH3_YEAST
332 LTV1_YEAST
333 PMG1_YEAST
334 KKQ8_YEAST
335 EBP2_YEAST
336 SN114_YEAST
337 MIA40_YEAST
338 YKT6_YEAST
339 XPOT_YEAST
340 LRC3_YEAST
341 UBA1_YEAST
342 VPS1_YEAST
343 PAP_YEAST
344 RSC4_YEAST
345 FOX2_YEAST
346 PRY2_YEAST
347 YKZ6_YEAST
348 YKR18_YEAST
349 GPT2_YEAST
350 YK54_YEAST;Q6Q573_YEAST
351 GTO2_YEAST
352 MTD1_YEAST
353 NU133_YEAST
354 SRP40_YEAST
355 BPT1_YEAST
356 SYDC_YEAST
357 HSP72_YEAST
358 HS104_YEAST
359 YL032_YEAST
360 PRP19_YEAST;Q8NJV2_YEAST
361 VPS13_YEAST
362 FPS1_YEAST
363 RL8B_YEAST
364 AATC_YEAST
365 PDC1_YEAST
366 GLYC_YEAST
367 SYFB_YEAST;Q6B2F2_YEAST

368 MED14_YEAST
369 IOC2_YEAST
370 KIN2_YEAST
371 Q05382_YEAST;MDN1_YEAST
372 YL108_YEAST
373 AHP1_YEAST
374 KICH_YEAST
375 PDC5_YEAST
376 STM1_YEAST
377 ACS2_YEAST
378 UBIQ_YEAST;RS37_YEAST;Q07188_YEAST;RL40_YEAST
379 METK1_YEAST
380 NOP56_YEAST
381 IFH1_YEAST
382 EF3A_YEAST
383 YL253_YEAST
384 GYS2_YEAST
385 HSP60_YEAST
386 DBP9_YEAST
387 YSH1_YEAST
388 YL287_YEAST
389 GSP1_YEAST
390 YL301_YEAST
391 RL38_YEAST
392 TMA10_YEAST
393 CHS5_YEAST
394 RS25B_YEAST;RS25A_YEAST
395 VRP1_YEAST;Q07229_YEAST
396 ILV5_YEAST;Q02340_YEAST;Q02341_YEAST
397 RS22B_YEAST;RS22A_YEAST
398 CCW14_YEAST
399 SKI2_YEAST
400 YL422_YEAST
401 CORO_YEAST
402 ECM30_YEAST
403 OAT_YEAST
404 RS3A_YEAST
405 FKBP4_YEAST
406 HMDH2_YEAST;Q6B2D0_YEAST
407 ERG6_YEAST
408 PPZ1_YEAST
409 SRC1_YEAST
410 RS3B_YEAST
411 DAK1_YEAST
412 TBA1_YEAST;TBA3_YEAST
413 ALO_YEAST
414 PHO84_YEAST
415 HMCS_YEAST
416 RSC9_YEAST
417 YMN1_YEAST;Q6Q5K5_YEAST
418 MVP1_YEAST
419 YMR7_YEAST
420 SUB1_YEAST
421 RNA14_YEAST
422 SEC14_YEAST

423 ADH3_YEAST
424 KU80_YEAST
425 ILVB_YEAST
426 MYO5_YEAST
427 PUR92_YEAST
428 PO152_YEAST
429 ALDH3_YEAST;ALDH2_YEAST
430 SSO2_YEAST;SSO1_YEAST
431 RL36A_YEAST
432 TOM40_YEAST
433 K6PF2_YEAST
434 SCJ1_YEAST
435 YM71_YEAST
436 RRP5_YEAST
437 RS10B_YEAST;RS10A_YEAST
438 RL20_YEAST
439 LCF4_YEAST
440 IF1A_YEAST
441 RSN1_YEAST
442 HAS1_YEAST
443 LCB1_YEAST
444 CBPY_YEAST
445 LIP1_YEAST
446 ATM1_YEAST
447 GAS1_YEAST
448 EIF3C_YEAST
449 PUB1_YEAST
450 HDA1_YEAST
451 SAM50_YEAST;Q6IEH7_YEAST
452 COG6_YEAST
453 VDAC1_YEAST
454 ARP5_YEAST
455 TRM6_YEAST
456 MAS5_YEAST
457 SUN4_YEAST
458 RL9B_YEAST
459 RL16B_YEAST
460 TPM1_YEAST
461
TOP2_YEAST;Q07114_YEAST;Q8TF86_YEAST;Q8TG43_YEAST;Q8TG44_YEAST;Q8TG46_YEAST;Q8TG47_YEAST;Q8TG53_YEAST;Q8TG56_YEAST;Q8TG58_YEAST
462 RS7B_YEAST;Q45TZ8_YEAST
463 RAS2_YEAST
464 LEU1_YEAST
465 DCP2_YEAST
466 TOM70_YEAST
467 CPT1_YEAST
468 FKBP_YEAST
469 CAP_YEAST
470 THO2_YEAST
471 LSM7_YEAST
472 YNO9_YEAST
473 KC12_YEAST
474 CBK1_YEAST
475 BNI5_YEAST

476 MDG1_YEAST
477 YNS1_YEAST
478 CHS1_YEAST
479 ALG9_YEAST
480 PURA_YEAST
481 PDR16_YEAST
482 RPA49_YEAST
483 SIP3_YEAST
484 DSL1_YEAST
485 PDR17_YEAST
486 COPG_YEAST
487 KRI1_YEAST
488 DUS2_YEAST
489 ACAC_YEAST
490 DBP6_YEAST
491 SIN3_YEAST
492 KCC2_YEAST;Q05436_YEAST
493 RLA2_YEAST
494 A6N9K9_YEAST;GSHB_YEAST
495 PSH1_YEAST
496 SYWC_YEAST;Q6B1Y2_YEAST
497 ZEO1_YEAST
498 HRP1_YEAST
499 IF4E_YEAST
500 RIB4_YEAST
501 NOP8_YEAST
502 ZPS1_YEAST
503 SHE4_YEAST
504 CSK22_YEAST
505 UFE1_YEAST
506 YVC1_YEAST
507 RPIA_YEAST
508 RS7A_YEAST
509 OST2_YEAST
510 INP53_YEAST
511 LEO1_YEAST
512 UBP2_YEAST
513 ORT1_YEAST
514 SMP3_YEAST
515 PDR5_YEAST
516 SYQ_YEAST
517 DCS2_YEAST
518 HEMH_YEAST
519 GSP2_YEAST
520 RPC2_YEAST
521 ODC2_YEAST
522 GRPE_YEAST
523 DGA1_YEAST
524 RPN8_YEAST
525 VPH1_YEAST
526 CAF20_YEAST
527 NOP58_YEAST
528 SYAC_YEAST
529 KRE5_YEAST
530 RPA1_YEAST

531 ETFD_YEAST
532 RS12_YEAST
533 DHE4_YEAST;DHE5_YEAST
534 ATF1_YEAST;Q6XBT2_YEAST
535 RRP12_YEAST
536 IRC15_YEAST
537 VTC3_YEAST
538 PMA2_YEAST
539 PDR12_YEAST
540 ALDH6_YEAST
541 TIM50_YEAST
542 MUK1_YEAST
543 ATPF_YEAST;Q6B1V4_YEAST
544 BRO1_YEAST
545 SEC16_YEAST
546 GSHR_YEAST
547 YP105_YEAST
548 HSP7F_YEAST
549 RNY1_YEAST
550 H1_YEAST
551 TBF1_YEAST
552 RL5_YEAST
553 RL33A_YEAST
554 OYE3_YEAST;Q6B154_YEAST
555 YP183_YEAST
556 UIP4_YEAST
557 RL7B_YEAST
558 SRP72_YEAST
559 BMS1_YEAST
560 FAS2_YEAST
561 RUVB2_YEAST
562 SRP68_YEAST
563 RL36B_YEAST
564 DIM1_YEAST
565 MDL2_YEAST
566 SAM3_YEAST
567 RPA2_YEAST
568 AGC1_YEAST
569 VATH_YEAST
570 TKT1_YEAST
571 TF2B_YEAST;Q6B148_YEAST
572 YP091_YEAST
573 COG4_YEAST
574 YLH47_YEAST
575 PLR1_YEAST
576 SCD6_YEAST
577 IWS1_YEAST
578 NOC4_YEAST
579 PHSG_YEAST
580 RHO1_YEAST
581 GDE_YEAST
582 SKI3_YEAST
583 RPC3_YEAST
