# Supplementary material for: Elevated Proteasome Capacity Extends Replicative Lifespan in Saccharomyces cerevisiae
Source: PLoS Genet. 2011 Sep 8;7(9):e1002253. doi: 10.1371/journal.pgen.1002253 (PMC3169524; doi:10.1371/journal.pgen.1002253)
Supplement: Table S7 — Primer Sequences. This table lists the primer sequences used for qRT-PCR experiments. (PDF) [file pgen.1002253.s010.pdf]

Table S7: Primer pairs for quantitative real-time PCR

Gene	Orientation	Primer Sequence
PRE1	Forward	CGTCTTCTAAGGCAGTCACAAG
PRE1	Reverse	CTTCACCGGCAAAACTCATC
RPN11	Forward	TGTTGCTGTCGTTGTTGACC
RPN11	Reverse	GCTTGAATGTTGGCCTTGTTC
PRE6	Forward	GCTAGAGTGGAGGCCCAAAG
PRE6	Reverse	TTGCACACCAGCGACATAAC
RPT2	Forward	AAAGTAGCGGGTGAGAATGC
RPT2	Reverse	GCAATTCCAGCATGGTTCTC
YAP1	Forward	CCA GGA AAT GAA AGC GAA ATC
YAP1	Reverse	ATC CCA AAT TTC CGA ACA CC
TRX2	Forward	TGG ATG TTG ATG AAG TCT CAG ATG
TRX2	Reverse	CCT CCT TAC CGC CCT TGT AG
TRR1	Forward	GCC ACA CTC CAG CAA CAA AG
TRR1	Reverse	ACA ACC AGA GCC AGC AGA AG
GRX2	Forward	AGG CCC TTG TGT TGG AAT TAG
GRX2	Reverse	TCG CTG TTA CCA CCA ATG TG
SOD2	Forward	CGT CAC AGG CCC ACT AGT TC
SOD2	Reverse	GAT CTT GCC AGC ATC GAA TC
